# Chirality and Rigidity in Triazole-Modified Peptidomimetics Interacting with Neuropilin-1

**DOI:** 10.3390/ph17020190

**Published:** 2024-01-31

**Authors:** Bartłomiej Fedorczyk, Patrycja Redkiewicz, Joanna Matalińska, Radosław Piast, Piotr Kosson, Rafał Wieczorek

**Affiliations:** 1 Faculty of Chemistry, University of Warsaw, Pasteura 1, 02-093 Warsaw, Poland; bfedorczyk@chem.uw.edu.pl (B.F.); rpiast@chem.uw.edu.pl (R.P.); 2 Mossakowski Medical Research Centre Polish Academy of Science, 5 Pawinskiego Street, 02-106 Warsaw, Poland; predkiewicz@imdik.pan.pl (P.R.); jmatalinska@imdik.pan.pl (J.M.); pkosson@imdik.pan.pl (P.K.)

**Keywords:** VEGF-165, neuropilin-1, peptidomimetics, triazole, structure–activity relationship

## Abstract

The interaction of Neuropilin-1 (NRP-1) with vascular endothelial growth factor (VEGF) has been shown to promote angiogenesis under physiological and pathological conditions. Angiogenesis around tumors is a major factor allowing for their growth and spread. Disrupting NRP-1/VEGF complex formation is thus a promising pathway for the development of new anticancer pharmaceuticals. A large body of work has been produced in the last two decades detailing the development of inhibitors of NRP-1/VEGF complex formation. Among those were peptide A7R and its smaller derivatives KXXR and K(Har)XXR. It has been previously reported that replacement of the XX backbone with triazole residues has a positive effect on the proteolytic stability of inhibitors. It has also been reported that a higher dihedral angle range restriction of the XX backbone has a positive effect on the activity of inhibitors. In this work, we have designed new triazole derivatives of K(Har)XXR inhibitors with substitution allowing for higher range restriction of the XX backbone. The obtained peptidomimetics have greater activity than their less restricted counterparts. One of the newly obtained structures has greater affinity than the reference peptide A7R.

## 1. Introduction

Compounds inhibiting the interaction between vascular endothelial growth factor 165 (VEGF_165_) and the complex of vascular endothelial growth factor receptor type-2 (VEGFR-2) with Neuropilin-1 (NRP-1) have been under intensive investigation in recent years [1,2,3,4,5,6,7,8,9,10,11,12,13,14,15,16,17,18,19,20]. The complex of these three proteins initiates the signaling leading to angiogenesis [21]. Angiogenesis is a process of new blood vessel formation from pre-existing vessels. It occurs as a result of local low oxygen levels in tissues, which itself is a byproduct of intensive tissue growth, for example in tissue development or healing [22,23]. An analogous situation occurs in intense tumor growth, which triggers vascularization through the same molecular pathways [24,25]. It is thus recognized since at least the 1990s that inhibiting the process of angiogenesis can be a potential anticancer strategy [25]. This recognition has sparked intense research into the inhibitors of the formation of this angionesis-inducing complex, especially the interaction between VEGF_165_ and Neuropilin-1 [1,2,3,4,5,6,7,8,9,10,11,12,13,14,15,16,17,18,19,20]. Neuropilin-1’s interaction with VEGF_165_ greatly enhances angiogenesis signaling, which can be also mediated solely by VEGFR-2 and VEGF_165_ [26]. Some cancers were even found to overexpress Neuropilin-1 as a mechanism that makes them more malignant [27,28,29].

Many of the currently investigated neuropilin inhibitors (“neuropilin inhibitor” will be used here as a shorthand for the inhibitor of the interaction between Neuropilin-1 and VEGF_165_) originate in their design from the initial discovery, through phage display method, of a 7-mer peptide of the sequence ATWLPPR (often referred to as A7R) with high potency against VEGF_165_/NRP-1 complex formation [30]. The shortest version of this peptide which retained high activity was the 4-mer LPPR [3]. It was also demonstrated that substitution of leucine into lysine, thus forming peptide KPPR, increased the inhibition of VEGF_165_ binding to NRP-1 [9,12]. The N- and C-terminal basic amino acids of this tetrapeptide are crucial for the affinity to the protein, while the second and third residues seem to be of structural importance [12,31]. The range of dihedral angles (omega, psi and fi) located in this two-residue-long backbone has been a subject of a dedicated study [14]. Since short peptides are known to exhibit a labile conformational character, specific methods of decreasing the conformation latitude in the KXXR structure could lead to a better inhibitor. It was found [14] that the more labile the backbone (XX in KXXR), the less active the analogues tetrapeptide. In another study, it was discovered that branching N-terminal lysine with additional homoarginine (Har) residue, affording Lys(Har)-Xaa-Xaa-Arg, results in more effective inhibition [17]. This scaffold, K(Har)XXR, has been subsequently used in further refinement. As it was observed that enzymatic cleavage of this structure occurred between the second and third residue (between XX) [17], its half-life in human body could be extended via the introduction of non-cleavable bioisosteres of peptide bonds in the XX region, thus producing a better therapeutic candidate [18].

Incorporation of 1,2,3-triazoles into biomimetic molecules is popular owing to the ease of preparing the click reaction through 1,3-dipolar cycloaddition catalyzed by copper ions [32,33]. The incorporation of a triazole ring into the peptide chain on the one hand changes the conformational latitude of the chain, and on the other it elongates the distance between the proceeding and subsequent residues by about 1 Å (3.9 Å for aa unit vs. 4.9 Å for triazole unit) on top of being proteolysis-resistant [34,35]. The XX residues in K(Har)XXR have been replaced with one or two 1,2,3-triazole units mimicking a glycine residue (coded as Gly[Trl]) [18]. A large number of compounds was thus obtained. All those compounds had improved proteolytic stability while still retaining their inhibitory activity. None of them, however, exhibited greater neuropilin inhibition than the A7R peptide. Among the most potent derivatives were those with two triazole substitutions, for example Lys(Har)-Gly[Trl]Gly[Trl]Arg, named in the present work **1L**. Molecular dynamics simulations of those compounds revealed their large flexibility [18], which was known from previous works to negatively correlate with inhibitory activity [14]. It was therefore postulated that further modifications introducing elements decreasing the conformational latitude of triazole moieties, e.g., branched amino acid derivatives instead of glycine derivatives (Gly[Trl]), may improve the affinity of the inhibitors to Neuropilin-1 [18].

In the present work, we have decided to test this hypothesis by synthesizing and examining a number of triazole derivatives of the K(Har)XXR structure, in which greater rigidity is introduced by additional methyl groups on Cα analogues of XX residues. Additionally, we have introduced variability in the chirality of the lysine moiety to explore different conformations in presumably more “restricted” structures.

## 2. Results

**Figure 1:**
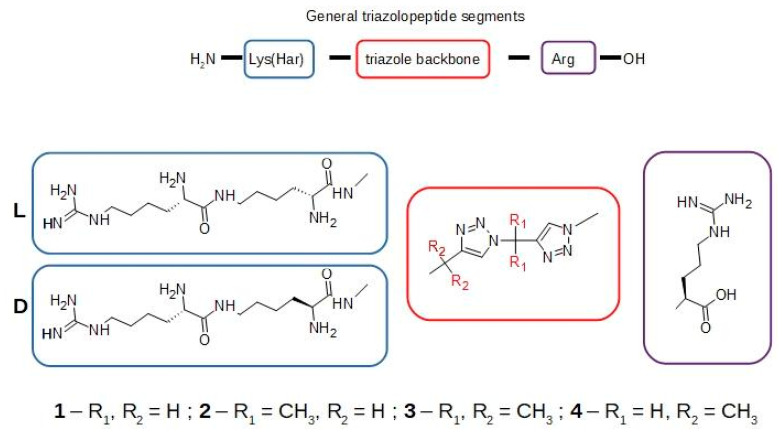
The general scheme of structures of compounds **1L**, **1D**, **2L**, **2D**, **3L**, **3D**, **4L** and **4D**.

We have designed several different triazole derivatives of the K(Har)XXR sequence. The compounds differ in the introduction or lack of methyl groups on the carbons neighboring the triazole rings. The scheme of the modifications is presented in Figure 1. The addition of methyl groups is expected to limit the rotational possibilities of the compounds once bound to the neuropilin protein. Variation in the chirality of the lysine residue allows for an exploration of greater conformational space by the more rigid structures and allows to obtain a highly stable molecule against proteolysis in human serum [18]. Both changes (chirality and rigidity) are expected to influence the inhibitory activity.

### 2.1. Synthesis

The designed compounds were synthesized using a standard 9-fluorenylmethoxycarbonyl (Fmoc) solid-phase peptide synthesis methodology, using standard polystyrene Wang resin preloaded with Fmoc-Arg(Pbf). The incorporation of an azide moiety as well as a subsequent Click Reaction were performed on solid support. Homoarginine residue was obtained through a guanylation reaction of the ε-amino group of lysine after Fmoc deprotection, also completely on solid support. Final cleavage from resin as well as subsequent purification were performed according to standard methods. Further details on the synthesis and purification can be found in the Materials and Methods section as well as in the previous literature [14,18]. Details of the guanidinylation reaction can be obtained in [17,36].

The purity of all synthesized compounds, as estimated using HPLC, were above 95%. The molecular weight of the compounds, confirming their molecular identity, was established with high-resolution mass spectrometry (HRMS). The full list of compounds with their sequences as well as calculated and measured masses together with their HPLC retention times is given in Table 1. All other synthesis data—structures of the compounds, HPLC chromatograms and HRMS spectra—are available in the Supplementary Material (Figures S1–S4).

### 2.2. Molecular Modeling

In silico models of new triazolopeptide analogues were based on our previous models of triazolopeptide-NRP-1 complexes described in [18]. Molecular dynamics simulations were performed within the Yasara package. After the alterations were introduced to the ligands, each model’s energy was minimalized (steepest descent) and molecular dynamics simulations were performed for 100 ns followed by another energy minimalization. This procedure was repeated three times for each ligand, and the model with the lowest energy was chosen for binding energy calculation. The binding energy for ligands was calculated with exclusion of solvent molecules. In the end, all the ligands were superposed and the root-mean-square deviation of atomic positions (RMSD) in relation to **1L** was calculated. Calculated RMSDs ranged from 0.3 Å to 2.6 Å and were higher for ligands with a dimethyl modification introduced between the lysine and triazole ring, suggesting that this change impacts the ligand–receptor binding the most. Additional residues of these compounds raise the binding energy due to non-ionic interaction with the protein. Since, however, these interactions are weak, the impact on binding seems to be small; there is no greater than 10% difference in the binding strength between the strongest and the weakest compound (Table 2). There is no clear binding correlation between L and D isomers.

**Figure 2:**
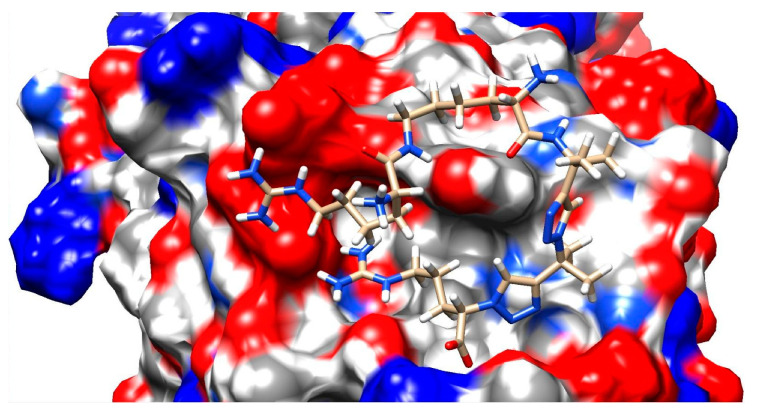
Complex of compound **3L** with NRP-1 after energy minimalization.

Full detailed values of the calculated energies are given in the Supplementary Materials in Table S1. Simulated structures of each compound docked to Neuropilin-1 can be found in Figure S8. The complex of compound **3L** with NRP-1 is presented in Figure 2.

**Figure 3:**
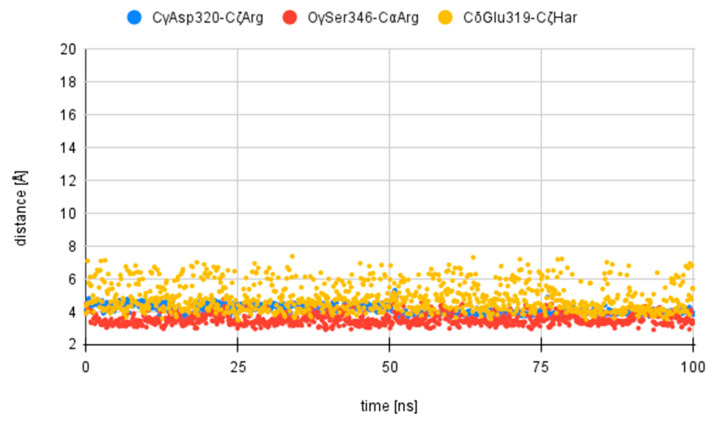
Calculated distances, in Å, between atoms from the **1L** compound and atoms of NRP-1 during 100 ns of simulation: blue—CγAsp320-CζArg; red—OγSer346-CαArg; yellow—CδGlu319-CζHar.

It has been previously suggested [14,18,37] that a number of atomic interactions are particularly important for the insight into structure–activity relationship of neuropilin inhibitors: ionic interaction of C-terminal Arg guanidine with Asp320 (CγAsp320-CζArg), hydrogen bonds of C-terminal Arg carboxylate with Ser346 (OγSer346-CαArg) and ionic interaction of Har guanidine with Glu319 (CδGlu319-CζHar). With this in mind, changes in the distances between those atoms have been monitored and plotted for the full duration of the simulation. In most cases, the interactions of the C-terminal arginine tended to show stable arrangement within the NRP-1 pocket, whereas the Har residue tended to more dynamically change its position vis à vis the surface of the protein. Figure 3 shows the distances for compound **1L**. A comparison of the obtained distances for all compounds is presented in the Supplementary Material (Figure S9).

### 2.3. Inhibitory Activity

In this work, a dedicated chemoluminescence affinity detection method was used. This method is reported to be more accurate than the previously standard competitive enzyme-linked immunosorbent assay (ELISA). The ELISA protocol was previously employed in [13,14,15,16,17,18]. See Materials and Methods and [19] for details on the chemoluminescence affinity detection method used here.

Briefly, the surfaces of flat-bottom polystyrene wells of 96-well plates were coated with human recombinant Neuropilin-1. The wells were then incubated with human biotinylated VEGF_165A_ {h(bt)VEGF} and subsequently with different concentrations of studied compounds, which then competitively bound to the NRP-1 detaching VEGF. The wells were then treated with streptavidin–horseradish peroxidase conjugate and with chemoluminescent substrate. Thus, the obtained color signal was directly proportional to the amount of bound biotinylated VEGF and therefore to the strength of the interaction of studied compounds with NRP-1 competing with VEGF. The strength of inhibition for different concentrations of different compounds is presented in Figure S7. IC_50_ was calculated as the concentration at which half of the bound VEGF was detached from NRP-1.

A summary of the strengths of NRP-1 binding as expressed by IC_50_ is presented in Table 2.

## 3. Discussion

A comparison of the inhibitors of the formation of the VEGF_165_/NRP-1 complex turns out to be surprisingly tricky. The A7R peptide has been the most studied molecule and the values of IC_50_ reported for it vary significantly; depending on the technique, assay, suppliers of reagents, laboratory and investigator, it has been reported to be 80 µM [30], 24 µM [1], 60 µM [2], 5.86 µM [13], 84 µM [19] and in the current work 31 µM (Figure S7). However, most of the works broadly agree by being within the same order of magnitude. Such a situation calls for attention and caution when reviewing and comparing the IC_50_ values published in different works [38]. On the other hand, it also creates an opportunity for an external standard. Studies of A7R should be encouraged to be performed alongside any new potential inhibitors. If one finds that the new inhibitor is two times weaker than A7R, it will give us rough estimation of its potency independently of the IC_50_ obtained for A7R.

The agreement between molecular modeling and the chemoluminescence inhibitory assay was rather poor. This might suggest that a more refined modeling is needed or that interaction resulting in competitive displacement of VEGF by incoming small molecules is more complex than simple affinity to the binding site. Also, changes in the distances between selected atoms of the inhibitors and the protein did not point to any clear-cut correlation. We can, however, observe some broad agreement between both methods. Additional methyl groups in the compound backbone give better affinity, either through limiting rotational freedom and thus limiting the time spent in non-optimal conformation as speculated before [18], or through better hydrophobic interactions with the protein. Also, neither methods detected any clear pattern regarding the impact of the chirality of the lysine moiety.

The major rationale for this study was the postulation that the greater rigidity of triazole-based peptidomimetics of the general structure K(Har)XXR will result in improved neuropilin inhibition. For this, we have designed several compounds in which the triazole analogue of glycine residue (Gly[Trl]) was replaced by one or two triazole 2-methyl-3-butyn-2-amine residues (Mba[Trl]). The introduction of two additional methyl groups for each replaced Gly[Trl] residue proved to be indeed beneficial. Previously, none of the triazolopeptides had activity greater than the reference peptide A7R. Compound **3L**, synthesized in the present work, in which two Gly[Trl] residues had been replaced with Mba[Trl] residues (Figure S3(La)), giving it greater rigidity among other properties, has been measured to possess an IC_50_ activity of 23 µM (Table 2, Figure S7(3,4)). This activity is markedly greater than that exhibited by the A7R peptide of 31 µM (Table 2, Figure S7). Also, compound **4D** displays similar activity to A7R at 36 µM (Table 2, Figure S7(3,4)). Combining that with its highly modified peptidomimetic structure (Figure S4(Da)) makes it another good pharmaceutical lead compound.

The insight gained in the previous works, especially [14,18], seems partially correct in its its emphasis on conformational latitude of XX residues in KXXR and K(Har)XXR inhibitors. However, the results obtained in the current work point towards more complex interactions, as evidenced by the pair **3L** and **3D**, whose IC_50_ is quite divergent, at 23 and 91 µM, respectively, despite the same levels of rigidity. Despite those reservations, the rationale behind the current work has led to the creation of molecules like **3L** and **4D**, possessing both high efficiency and proteolysis-resistant backbones, thus being perfect candidates for lead compounds for the development of therapeutically useful drugs for the suppression of angiogenesis in tumors and other conditions.

## 4. Materials and Methods

### 4.1. General Synthesis

Solvents were purchased from Iris Biotech, Marktredwitz, Germany (DMF, DCM) or Sigma-Aldrich, Burlington, MA, USA (MeOH, Et2O, THF). THF was distilled before use. Other solvents were used without further purification. Fmoc-protected aminoacids ((Boc-Lys(Fmoc)-OH, Boc-D-Lys(Fmoc)-OH and Fmoc-Har(Pbf)-OH), TFA and TIS (triisopropylsilane) were purchased from Iris Biotech. Coupling reagents as well as preloaded resin Fmoc-Arg(Pbf) with 0.55 mmol/g loading were obtained from Activotec, Cambridge, UK. Propargylamine, 2-Methyl-3-butyn-2-amine and N-(9H-fluoren-9-ylmethoxycarbonyloxy) succinimide (Fmoc-OSu) were purchased from Sigma-Aldrich. Imidazole-1-sulfonyl azide hydrochloride was provided by Trimen Chemicals, Łódź, Poland.

All researched compounds **1L**, **1D**, **2L**, **2D**, **3L**, **3D**, **4L** and **4D** were obtained manually using solid-phase peptide synthesis according to standard procedures employed for the Fmoc strategy [37]. All presented general procedures are described for typical synthesis at 0.1 mmol scale.

#### 4.1.1. Fmoc Deprotection

A typical two-step Fmoc deprotection reaction with the use of 20% piperidine in DMF was applied. In the first step, peptidylresin was treated with the solution for 5 min and further for 20 min with a fresh portion of the solution. Afterwards, peptidylresin was flushed with DMF and 2-propanol alternately (3 times each), and then 3 times with DMF.

#### 4.1.2. Monitoring of the Reaction Progress

For detection of the presence of the amine groups located on the peptidylresin, a chloranil test was used. Briefly, 20 μL of solution A (2% chroranil in DMF) and 20 μL of solution B (2% acetaldehyde in DMF) were placed in an Eppendorf tube and a small portion of peptidylresin was placed in the solution for 5 min of incubation at room temperature. Afterwards, the color of the beads was inspected on a white background. Firstly, beads turned orange, and after several minutes a greenish color appeared—showing the presence of amine groups on peptidyl resin—or it turned colorless—showing the absence of the amine groups on peptidyl resin. The test also allows for the detection of additional primary amino groups that lack an alpha-hydrogen atom.

#### 4.1.3. Wong Diazotransfer

During a typical reaction procedure, a portion of 2 eq. (0.2 mmol, 42 mg) of Imidazole-1-sulfonyl azide hydrochloride was diluted in 1.5 mL of methanol. Afterwards it was further diluted with DCM (8 mL). Then, 0.75 mL of an aqueous solution containing 4 eq. of K_2_CO_3_ (0.4 mmol, 55 mg) and 0.75 mL of an aqueous solution containing 0.02 eq. (0.002 mmol, 0.5 mg) of copper(II) sulfate pentahydrate were added to the mixture. After preshaking, the mixture was sucked into a luer-locked syringe with peptydylresin. Mixture was shaken constantly during the reaction, which was left to proceed overnight. Afterwards, peptidylresin was washed four times with DCM. Completion of the reaction was confirmed with a negative chloranil test.

#### 4.1.4. Fmoc-Propargylamine (5) and Fmoc-Mba (6) Preparation

Fmoc-Propargylamine and Fmoc-Mba (Fmoc-2-methyl-3-butyn-2-amine) were prepared accordingly as described before [18]. The structures of both compounds, as well as the spectra of their ^1^H and ^13^C NMR analysis, can be found in the Supplementary Materials (Figures S5 and S6). ^1^H and ^13^C NMR spectra were recorded on a Bruker 500 MHz spectrometer (Bruker Corporation, Billerica, MA, USA).

Fmoc-Propargylamine (**5**): 1H NMR (DMSO-d6, 500 MHz) δ 7.89 (2H, d, *J* = 7.5 Hz, Fmoc H4 and H5), 7.78 (1H, t, *J* = 5.5 Hz, NH), 7.69 (2H, d, *J* = 7.0 Hz, Fmoc H1 and H8), 7.41 (2H, td, *J* = 7.5, 0.5 Hz, Fmoc H3 and H6), 7.33 (2H, td, *J* = 7.5, 1.0 Hz, Fmoc H2 and H7), 4.32 (2H, d, *J* = 7.0 Hz, Fmoc CH2), 4.22 (1H, t, *J* = 6.7 Hz, Fmoc H9), 3.78 (2H, dd, *J* = 6.0, 2.5 Hz, H3), 3.11 (1H, t, *J* = 2.5 Hz, H1);

13C NMR (DMSO-d6, 125 MHz) δ 155.92 (C), 143.81 (C), 143.81 (C), 140.73 (C), 140.73 (C), 127.64 (CH), 127.64 (CH), 127.08 (CH), 127.08 (CH), 125.15 (CH), 125.15 (CH), 120.13 (CH), 120.13 (CH), 81.38 (C), 73.05 (CH), 65.67 (CH2), 46.61 (CH), 29.78 (CH2).

Fmoc-Mba (**6**): 1H NMR (DMSO-d6, 500 MHz) δ 7.89 (2H, d, *J* = 7.5 Hz, Fmoc H4 and H5), 7.73 (2H, d, *J* = 7.5 Hz, Fmoc H1 and H8), 7.58 (1H, s, NH), 7.41 (2H, td, *J* = 7.5, 0.5 Hz, Fmoc H3 and H6), 7.33 (2H, td, *J* = 7.5, 1.0 Hz, Fmoc H2 and H7), 4.27 (2H, d, *J* = 5.5 Hz, Fmoc CH2), 4.20 (1H, t, *J* = 6.5 Hz, Fmoc H9), 3.07 (1H, s, H1), 1.47 (6H, s, Me groups);

13C NMR (DMSO-d6, 125 MHz) δ 154.37 (C), 143.90 (C), 143.90 (C), 140.72 (C), 140.72 (C), 127.62 (CH), 127.62 (CH), 127.06 (CH), 127.06 (CH), 125.28 (CH), 125.28 (CH), 120.10 (CH), 120.10 (CH), 88.10 (C), 88.10 (C), 70.77 (CH), 65.19 (CH2), 46.68 (CH3), 46.13 (CH3), 29.14 (C).

#### 4.1.5. Click Reaction on Solid Support

This step proceeded in accordance with previous descriptions [18,33]. Briefly, a portion of Fmoc-protected alkylamine (3 eq., 0.3 mmol) was dissolved in 6 mL of THF. Afterwards, 1 mL of aqueous solution containing 0.02 eq. (0.002 mmol, 0.5 mg) copper (II) sulfate was added to the mixture, which was sucked into a reaction syringe. Next, a portion of sodium ascorbate (20 mg, 0.1 mmol) was dissolved in 1 mL w MiliQ water and separately sucked into a reaction syringe with peptydylresin. The reaction was left to complete overnight. Afterwards, peptidylresin was washed four times with THF and then Fmoc-deprotected. Further, a positive result for the chloranil test was observed.

#### 4.1.6. Common Procedures for Peptide Chain Elongation on Solid Support

For the amino acid coupling reaction, a standard protocol was used. Briefly, 3 eq. of OxymaPure and 3 eq. of Fmoc-protected amino acid were dissolved in 6 mL of DMF. Afterwards, a portion of 3 eq. of DIC was added to the mixture and mixed with peptidylresin for 1 h. Completion of the reaction was inspected with a chloranil test.

#### 4.1.7. Peptidotriazole Cleavage from Solid Support

Resin with the final product was washed three times with DMC, three times with MeOH, three times with Et_2_O and dried in a desiccator under reduced pressure. For final deprotection and cleavage from solid support, the resin was treated with a mixture of TFA/TIS/H2O (0.95:2.5:2.5 in volume ratio) for 3 h. After filtration and three washings of the resin with additional TFA, the combined supernatant was concentrated on a rotary evaporator. The residue was diluted with cold Et_2_O (−20 °C) and the precipitated product was collected through centrifuging and decanting.

#### 4.1.8. HPLC Analysis, Purification and HRMS Characterization

Crude peptidotriazoles were analyzed using RP-HPLC analytical column Jupiter Proteo I.D. 4.6 mm × 250 mm and purified on preparative column Jupiter Proteo I.D. 21.2 mm × 250 mm. Phase A: miliQ water + 0.1% TFA (*v*/*v*); phase B: HPLC-grade acetonitrile + 0.1% TFA (*v*/*v*). Elution program for all compounds: linear increase in phase B from 1% to 21% in 20 min. Detection was carried out at 220 nm. Analytical flow = 1 mL/min; preparative flow = 20 mL/min. Both were performed on the Shimadzu Prominence system. Fractions containing pure peptidotriazoles were freeze-dried. Pure compounds were analyzed using high-resolution mass spectrometry to confirm their general formula. High-resolution mass spectra were acquired on the Shimadzu LCMS-IT-TOF mass spectrometer on Phenomenex Jupiter 4u Proteo 90 Å C12 (25 cm × 2 mm × 4 µm) column (Shimadzu LCMS-2010EV (Kyoto, Japan) mass spectrometer with electrospray ionization).

### 4.2. Molecular Dynamics

We have based new in silico structure models of triazolopeptide-NRP-1 complexes on our previous model [18]. New analogs were generated in Yasara Strucure 20.8.23. The structure of the NRP-1 protein was taken from the Protein Data Bank, accession number 2ORZ [39]. Molecular dynamics simulations were performed within the Yasara package. After each molecule was generated, the energy of the complex was minimized using the steepest descent algorithm. Subsequently, molecular dynamics simulations were performed using the Amber ipq15 force field [40] with periodic boundary conditions, default simulation cell size and water molecules as solvent (density 0.997 g/mL) with an ionic strength equal to saline, and temperature 303.15 K. The protonation states (in ligands and the protein) were set as pH = 7.

Each triazolopeptide analogue was subjected to molecular dynamics in 3 independent runs lasting 100 ns with a simulation snapshot every 0.1 ns. At the end of each simulation, the energy was once again minimized and the binding energy was calculated between the ligands and NRP-1 protein. Structures with the highest binding energy were chosen for the comparison. In the end, all ligands were superimposed and the root-mean-square deviation of atomic positions (RMSD) in relation to compound **1L** was calculated. The relative binding energy of compound **1L** was set to 100% (Table 2 and Table S1).

Additionally, changes in the distances between the Cγ atom of Asp320 and Cζ of the Arg residue of the studied compounds (**1L,D** to **4L,D**), the Oγ atom of Ser346 and Cα of the Arg residue and the Cδ atom of Glu319 and Cζ of the Har residue were plotted.

### 4.3. ELISA Assays of Inhibitory Activity

The flat-bottom wells of polystyrene microplates (Nunc Immuno™ MicroWell™ 96 well solid plates) were coated overnight at 4 °C with recombinant human NRP-1 (hNRP-1) (BioLegend, San Diego, CA, USA) at 2 µg/mL. Then, the wells were washed twice with 0.5% Tween20 phosphate-buffered saline (PBS) and subsequently incubated for 2 h at RT with 200 µL of 10.5% BSA in PBS in order to block unspecific binding. The plates were then rinsed with distilled water and were subsequently incubated for 2 h at RT with 50 µL (400 ng/mL) of h(bt)VEGF 165 A (Abcam, Cambridge, UK) in PBS with 4 µg/mL heparine as well as with 50 µL of the studied compounds in PBS in different concentrations (12.5 µM, 25 µM, 50 µM and 100 µM). The plates were rinsed again with distilled water and incubated for 45 min at RT in 100 µL of streptavidin–horseradish peroxidase conjugate in PBS. After one more water rinse, 100 μL chemiluminescent substrate was added. The luminescence was quantified immediately after addition of the substrate. The percentages of inhibition were calculated with the formula 100% − {(S − SN)/(P − NS)} × 100%. S is the intensity of the measured signal, NS is the intensity of the measured signal in the negative control and P is the intensity of the measured signal in the positive control. The positive control was incubated solely with VEGF 165A with no studied compound. Negative control was obtained from wells which were not coated with hNRP-1. All experiments were performed in triplicate. Determination of IC_50_ was carried out using the nonlinear regression function with Prism (Version 10.1.2, GraphPad software). The standard (fitting) error of the mean was calculated using Prism’s Sy.x function.

Further details about this assay can be found in [19].

## Figures and Tables

**Table 1:** Characterization of synthesized compounds.

No.	Sequence	RT [min]	Cal MS [*m/z*]	Meas. MS [*m/z*]	Difference [ppm]
**1L**	H-Lys(Har)-Gly[Trl]Gly[Trl]Arg-OH	12.65	[M+H]+ 635.3961	[M+H]+ 635.3935	4.09
**1D**	H-D-Lys(Har)-Gly[Trl]Gly[Trl]Arg-OH	12.73	[M+H]+ 635.3961	[M+H]+ 635.3944	2.68
**2L**	H-Lys(Har)-Gly[Trl]Mba[Trl]Arg-OH	15.36	[M+H]+ 663.4274	[M+H]+ 663.4260	2.11
**2D**	H-D-Lys(Har)-Gly[Trl]Mba[Trl]Arg-OH	15.37	[M+H]+ 663.4274	[M+H]+ 663.4268	0.90
**3L**	H-Lys(Har)-Mba[Trl]Mba[Trl]Arg-OH	17.58	[M+H]+ 691.4587	[M+H]+ 691.4577	1.45
**3D**	H-D-Lys(Har)-Mba[Trl]Mba[Trl]Arg-OH	17.72	[M+H]+ 691.4587	[M+H]+ 691.4573	2.02
**4L**	H-Lys(Har)-Mba[Trl]Gly[Trl]Arg-OH	15.20	[M+H]+ 663.4274	[M+H]+ 663.4261	1.96
**4D**	H-D-Lys(Har)-Mba[Trl]Gly[Trl]Arg-OH	15.15	[M+H]+ 663.4274	[M+H]+ 663.4273	0.15

**Table 2:** Simulated energies of NRP-1 binding and measured half-maximal inhibitory concentrations of VEGF 165A displacement from NRP-1 by the studied compounds.

No.	Relative Binding Energy	IC_50_
**1L**	100.0%	68.5 ± 3.3 µM
**1D**	96.1%	63.6 ± 2.3 µM
**2L**	100.3%	87.5 ± 3.3 µM
**2D**	101.4%	69.4 ± 4.0 µM
**3L**	105.3%	23.4 ± 1.3 µM
**3D**	106.7%	90.5 ± 6.2 µM
**4L**	101.3%	>100.0 ± 14.6 µM
**4D**	102.3%	35.6 ± 4.5 µM
A7R	-	31.2 ± 4.2 µM

## Data Availability

Data are contained within the article and Supplementary Materials.

## Supplementary Materials

The following supporting information can be downloaded at https://www.mdpi.com/article/10.3390/ph17020190/s1: Figures S1–S4: Structure, HPLC chromatogram and HMRS spectra of compounds **1L,D** to **4L,D**; Figures S5 and S6: Structure and ^1^H and ^13^C NMR spectra of compounds **5** and **6**; Figure S7: Inhibition of VEGF_165_ binding to human NRP-1 by A7R peptide and compounds **1L,D** to **4L,D**; Table S1: Details of molecular modeling; Figure S8: Illustration of complexes of compounds **1L,D** to **4L,D** with Neuropilin-1; Figure S9: Calculated distances between selected atoms of the studied compounds (**1L,D** to **4L,D**) and selected atoms of NRP-1.

## References

[1] Perret G.Y., Starzec A., Hauet N., Vergote J., Le Pecheur M., Vassy R., Léger G., Verbeke K.A., Bormans G., Nicolas P. (2004). In vitro evaluation and biodistribution of a 99mTc-labeled anti-VEGF peptide targeting neuropilin-1. Nucl. Med. Biol..

[2] Starzec A., Vassy R., Martin A., Lecouvey M., Di Benedetto M., Crépin M., Perret G.Y. (2006). Antiangiogenic and antitumor activities of peptide inhibiting the vascular endothelial growth factor binding to neuropilin-1. Life Sci..

[3] Starzec A., Ladam P., Vassy R., Badache S., Bouchemal N., Navaza A., du Penhoat C.H., Perret G.Y. (2007). Structure–function analysis of the antiangiogenic ATWLPPR peptide inhibiting VEGF_165_ binding to neuropilin-1 and molecular dynamics simulations of the ATWLPPR/neuropilin-1 complex. Peptides.

[4] Starzec A., Miteva M.A., Ladam P., Villoutreix B.O., Perret G.Y. (2014). Discovery of novel inhibitors of vascular endothelial growth factor-A–Neuropilin-1 interaction by structure-based virtual screening. Bioorg. Med. Chem..

[5] Thomas N., Pernot M., Vanderesse R., Becuwe P., Kamarulzaman E., Da Silva D., François A., Frochot C., Guillemin F., Barberi-Heyob M. (2010). Photodynamic therapy targeting neuropilin-1: Interest of pseudopeptides with improved stability properties. Biochem. Pharmacol..

[6] Novoa A., Pellegrini-Moïse N., Bechet D., Barberi-Heyob M., Chapleur Y. (2010). Sugar-based peptidomimetics as potential inhibitors of the vascular endothelium growth factor binding to neuropilin-1. Bioorg. Med. Chem..

[7] Richard M., Chateau A., Jelsch C., Didierjean C., Manival X., Charron C., Maigret B., Barberi-Heyob M., Chapleur Y., Boura C. (2016). Carbohydrate-based peptidomimetics targeting neuropilin-1: Synthesis, molecular docking study and in vitro biological activities. Bioorg. Med. Chem..

[8] Borriello L., Montès M., Lepelletier Y., Leforban B., Liu W.-Q., Demange L., Delhomme B., Pavoni S., Jarray R., Boucher J.L. (2014). Structure-based discovery of a small non-peptidic Neuropilins antagonist exerting in vitro and in vivo anti-tumor activity on breast cancer model. Cancer Lett..

[9] Liu W.-Q., Megale V., Borriello L., Leforban B., Montès M., Goldwaser E., Gresh N., Piquemal J.-P., Hadj-Slimane R., Hermine O. (2014). Synthesis and structure–activity relationship of non-peptidic antagonists of neuropilin-1 receptor. Bioorg. Med. Chem. Lett..

[10] Liu W.-Q., Borriello L., Allain B., Pavoni S., Lopez N., Hermine O., Garbay C., Raynaud F., Lepelletier Y., Demange L. (2015). New Peptides Structurally Related to VEGF-A_165_ Exon-7 and-8 Encoded Domains Antagonize Its Binding to NRP-1 and VEGF-R1. Int. J. Pept. Res. Ther..

[11] Jia H., Bagherzadeh A., Hartzoulakis B., Jarvis A., Löhr M., Shaikh S., Aqil R., Cheng L., Tickner M., Esposito D. (2006). Characterization of a bicyclic peptide neuropilin-1 (NP-1) antagonist (EG3287) reveals importance of vascular endothelial growth factor exon 8 for NP-1 binding and role of NP-1 in KDR signaling. J. Biol. Chem..

[12] Jarvis A., Allerston C.K., Jia H., Herzog B., Garza-Garcia A., Winfield N., Ellard K., Aqil R., Lynch R., Chapman C. (2010). Small molecule inhibitors of the neuropilin-1 vascular endothelial growth factor A (VEGF-A) interaction. J. Med. Chem..

[13] Grabowska K., Puszko A.K., Lipiński P.F., Laskowska A.K., Wileńska B., Witkowska E., Misicka A. (2016). Design, synthesis and in vitro biological evaluation of a small cyclic peptide as inhibitor of vascular endothelial growth factor binding to neuropilin-1. Bioorg. Med. Chem. Lett..

[14] Fedorczyk B., Lipiński P.F., Tymecka D., Puszko A.K., Wilenska B., Perret G.Y., Misicka A. (2017). Conformational latitude–activity relationship of KPPR tetrapeptide analogues toward their ability to inhibit binding of vascular endothelial growth factor 165 to neuropilin-1. J. Pept. Sci..

[15] Grabowska K., Puszko A.K., Lipiński P.F., Laskowska A.K., Wileńska B., Witkowska E., Perret G.Y., Misicka A. (2017). Structure-activity relationship study of a small cyclic peptide Hc [Lys-Pro-Glu]-Arg-OH: A potent inhibitor of Vascular Endothelial Growth Factor interaction with Neuropilin-1. Bioorg. Med. Chem..

[16] Tymecka D., Lipiński P.F., Fedorczyk B., Puszko A., Wileńska B., Perret G.Y., Misicka A. (2017). Structure-activity relationship study of tetrapeptide inhibitors of the Vascular Endothelial Growth Factor A binding to Neuropilin-1. Peptides.

[17] Tymecka D., Puszko A.K., Lipiński P.F., Fedorczyk B., Wilenska B., Sura K., Perret G.Y., Misicka A. (2018). Branched pentapeptides as potent inhibitors of the vascular endothelial growth factor 165 binding to Neuropilin-1: Design, synthesis and biological activity. Eur. J. Med. Chem..

[18] Fedorczyk B., Lipiński P.F.J., Puszko A.K., Tymecka D., Wilenska B., Dudka W., Perret G.Y., Wieczorek R., Misicka A. (2019). Triazolopeptides inhibiting the interaction between neuropilin-1 and vascular endothelial growth factor-165. Molecules.

[19] Puszko A.K., Sosnowski P., Tymecka D., Raynaud F., Hermine O., Lepelletier Y., Misicka A. (2019). Neuropilin-1 peptide-like ligands with proline mimetics, tested using the improved chemiluminescence affinity detection method. Medchemcomm.

[20] Puszko A.K., Sosnowski P., Rignault-Bricard R., Hermine O., Hopfgartner G., Pułka-Ziach K., Lepelletier Y., Misicka A. (2020). Urea-Peptide Hybrids as VEGF-A_165_/NRP-1 Complex Inhibitors with Improved Receptor Affinity and Biological Properties. Int. J. Mol. Sci..

[21] Fong G.H., Rossant J., Gertsenstein M., Breitman M.L. (1995). Role of the Flt-1 receptor tyrosine kinase in regulating the assembly of vascular endothelium. Nature.

[22] Tonnesen M.G., Feng X., Clark R.A. (2000). Angiogenesis in wound healing. Journal of Investigative Dermatology Symposium Proceedings.

[23] Semenza G.L. (2007). Regulation of tissue perfusion in mammals by hypoxia-inducible factor 1. Exp. Physiol..

[24] Shubik P. (1982). Vascularization of tumors: A review. J. Cancer Res. Clin. Oncol..

[25] Folkman J. (1995). Angiogenesis in cancer, vascular, rheumatoid and other disease. Nat. Med..

[26] Soker S., Miao H.Q., Nomi M., Takashima S., Klagsbrun M. (2002). VEGF_165_ mediates formation of complexes containing VEGFR-2 and neuropilin-1 that enhance VEGF_165_-receptor binding. J. Cell. Biochem..

[27] Fakhari M., Pullirsch D., Abraham D., Paya K., Hofbauer R., Holzfeind P., Hofmann M., Aharinejad S. (2002). Selective upregulation of vascular endothelial growth factor receptors neuropilin-1 and-2 in human neuroblastoma. Cancer.

[28] Pallaoro A., Braun G.B., Moskovits M. (2011). Quantitative ratiometric discrimination between noncancerous and cancerous prostate cells based on neuropilin-1 overexpression. Proc. Natl. Acad. Sci. USA.

[29] Kreuter M., Woelke K., Bieker R., Schliemann C., Steins M., Buechner T., Berdel W.E., Mesters R.M. (2006). Correlation of neuropilin-1 overexpression to survival in acute myeloid leukemia. Leukemia.

[30] Binétruy-Tournaire R., Demangel C., Malavaud B., Vassy R., Rouyre S., Kraemer M., Plouët J., Derbin C., Perret G., Mazié J.C. (2000). Identification of a peptide blocking vascular endothelial growth factor (VEGF)-mediated angiogenesis. EMBO J..

[31] Teesalu T., Sugahara K.N., Kotamraju V.R., Ruoslahti E. (2009). C-end rule peptides mediate neuropilin-1-dependent cell, vascular, and tissue penetration. Proc. Natl. Acad. Sci. USA.

[32] Tornøe C.W., Christensen C., Meldal M. (2002). Peptidotriazoles on solid phase: [1,2,3]-triazoles by regiospecific copper (I)-catalyzed 1, 3-dipolar cycloadditions of terminal alkynes to azides. J. Org. Chem..

[33] Wamberg M.C., Wieczorek R., Brier S.B., de Vries J.W., Kwak M., Herrmann A., Monnard P.A. (2014). Functionalization of Fatty Acid Vesicles through Newly Synthesized Bolaamphiphile–DNA Conjugates. Bioconjug. Chem..

[34] Salah K.B.H., Das S., Ruiz N., Andreu V., Martinez J., Wenger E., Amblard M., Didierjean C., Legrand B., Inguimbert N. (2018). How are 1, 2, 3-triazoles accommodated in helical secondary structures?. Org. Biomol. Chem..

[35] Diness F., Schoffelen S., Meldal M. (2015). Advances in merging triazoles with peptides and proteins. Peptidomimetics I.

[36] Bernatowicz M.S., Wu Y., Matsueda G.R. (1992). 1H-Pyrazole-1-carboxamidine hydrochloride an attractive reagent for guanylation of amines and its application to peptide synthesis. J. Org. Chem..

[37] Behrendt R., White P., Offer J. (2016). Advances in Fmoc solid-phase peptide synthesis. J. Pept. Sci..

[38] Peng K., Bai Y., Zhu Q., Hu B., Xu Y. (2019). Targeting VEGF–neuropilin interactions: A promising antitumor strategy. Drug Discov. Today.

[39] Vander Kooi C.W., Jusino M.A., Perman B., Neau D.B., Bellamy H.D., Leahy D.J. (2007). Structural basis for ligand and heparin binding to neuropilin B domains. Proc. Natl. Acad. Sci. USA.

[40] Debiec K.T., Cerutti D.S., Baker L.R., Gronenborn A.M., Case D.A., Chong L.T. (2016). Further along the road less traveled: AMBER ff15ipq, an original protein force field built on a self-consistent physical model. J. Chem. Theory Comput..

