# Supplementary material for: Chirality and Rigidity in Triazole-Modified Peptidomimetics Interacting with Neuropilin-1
Source: Pharmaceuticals (Basel). 2024 Jan 31;17(2):190. doi: 10.3390/ph17020190 (PMC10891769; doi:10.3390/ph17020190)
Supplement: Supplementary file 1 [file pharmaceuticals-17-00190-s001.zip › pharmaceuticals-2768992-supplementary.pdf]

Supplementary Materials to:

Chirality and rigidity in triazole-modified peptidomimetics interacting with Neuropilin-1

by

Bartłomiej Fedorczyk, Patrycja Redkiewicz, Joanna Matalińska, Radosław Piast, Piotr Kosson and Rafał Wieczorek *

***Corresponding authors:** Rafał Wieczorek, wieczorek@chem.uw.edu.pl,

Table of contents

Synthesis data 2
Inhibitory activity 17
Molecular Modeling 20

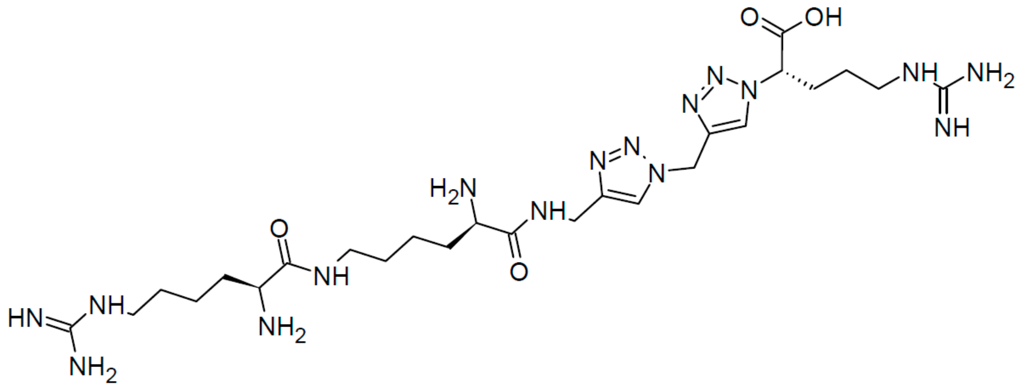

Figure S-1La. Structure of compound **1L** H-Lys(Har)-Gly[Trl]Gly[Trl]Arg-OH.

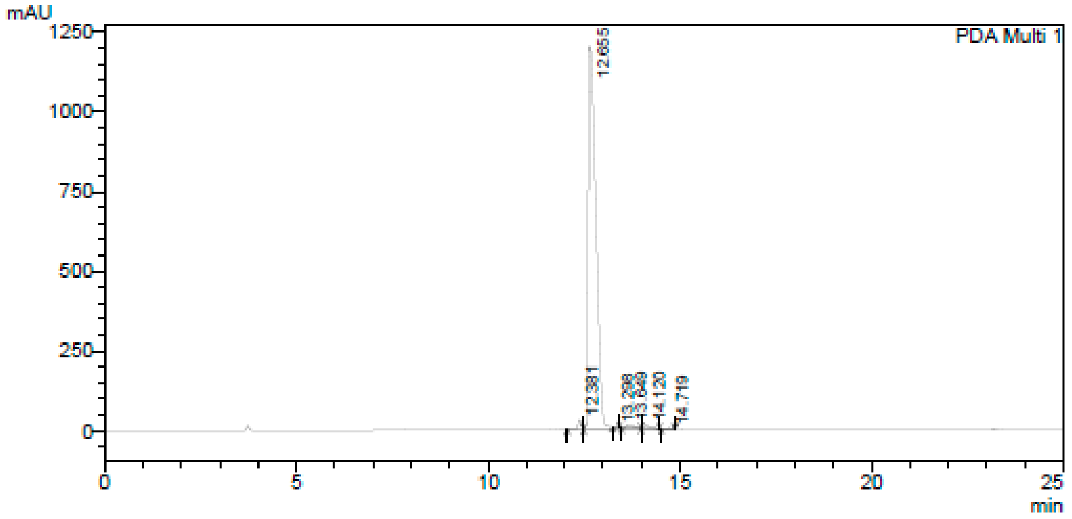

Figure S-1Lb. HPLC chromatogram of compound **1L** at 220 nm.

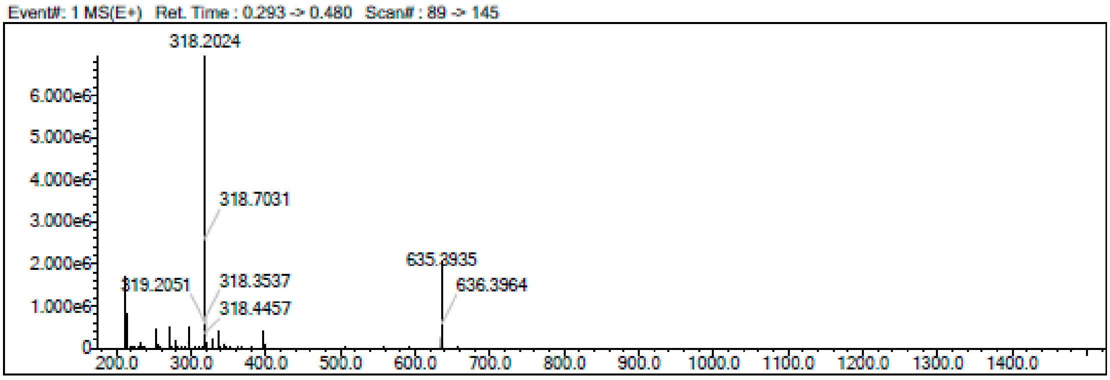

Figure S-1Lc. MS analysis of compound **1L**.

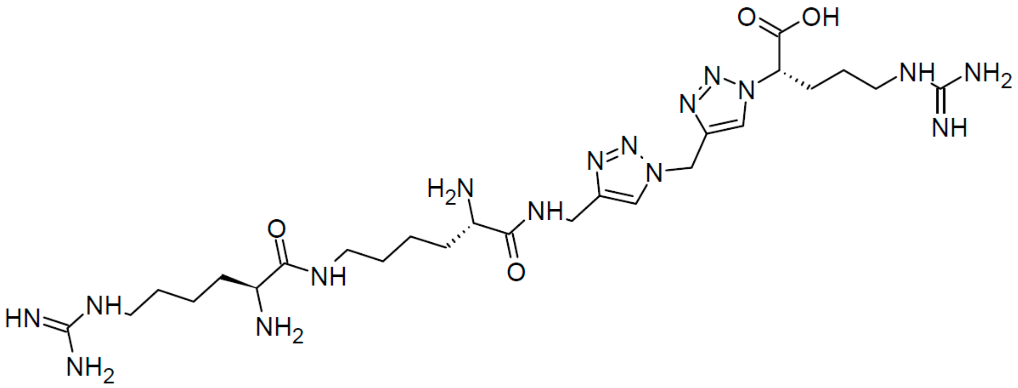

Figure S-1Da. Structure of compound **1D** H-D-Lys(Har)-Gly[Trl]Gly[Trl]Arg-OH.

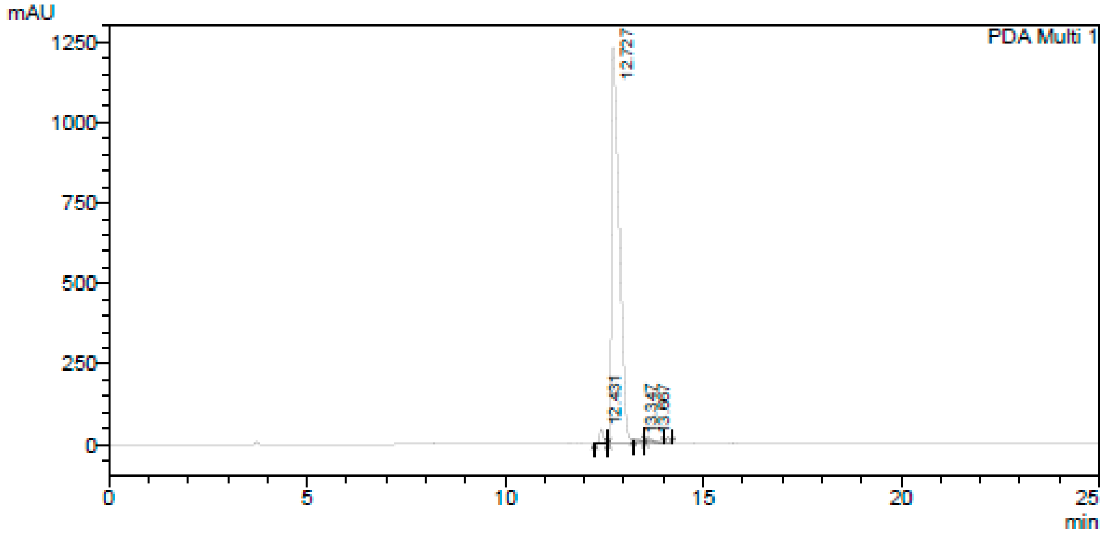

Figure S-1Db. HPLC chromatogram of compound **1D** at 220 nm.

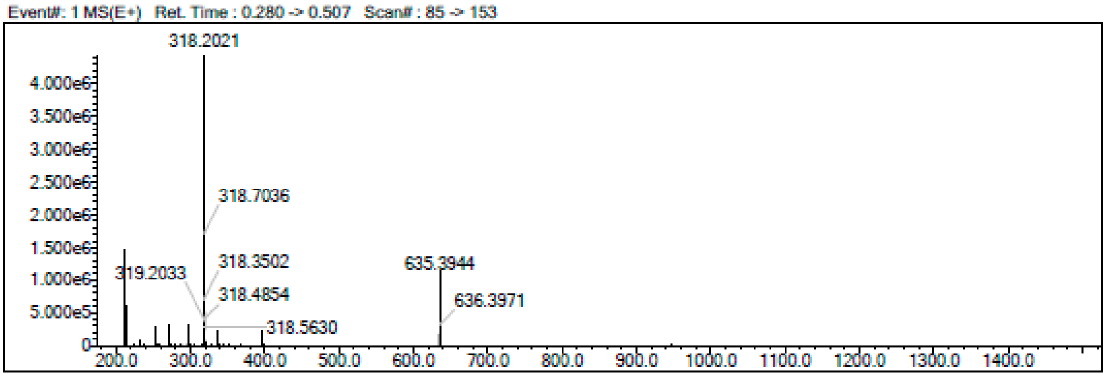

Figure S-1Dc. MS analysis of compound **1D**.

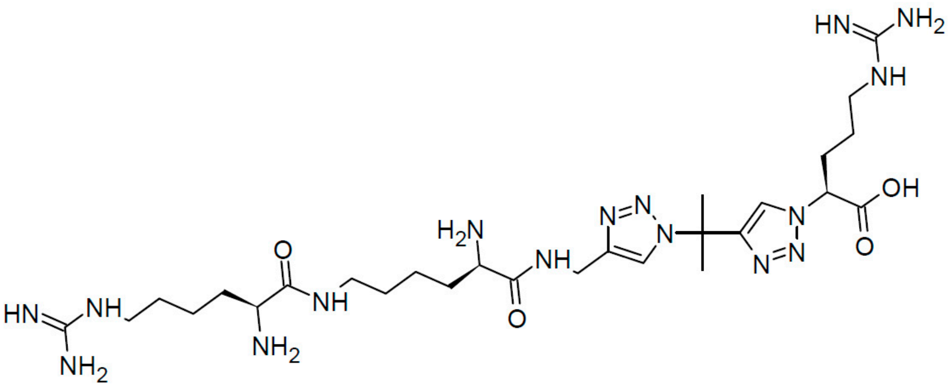

Figure S-2La. Structure of compound **2L** H-Lys(Har)-Gly[Trl]Mba[Trl]Arg-OH.

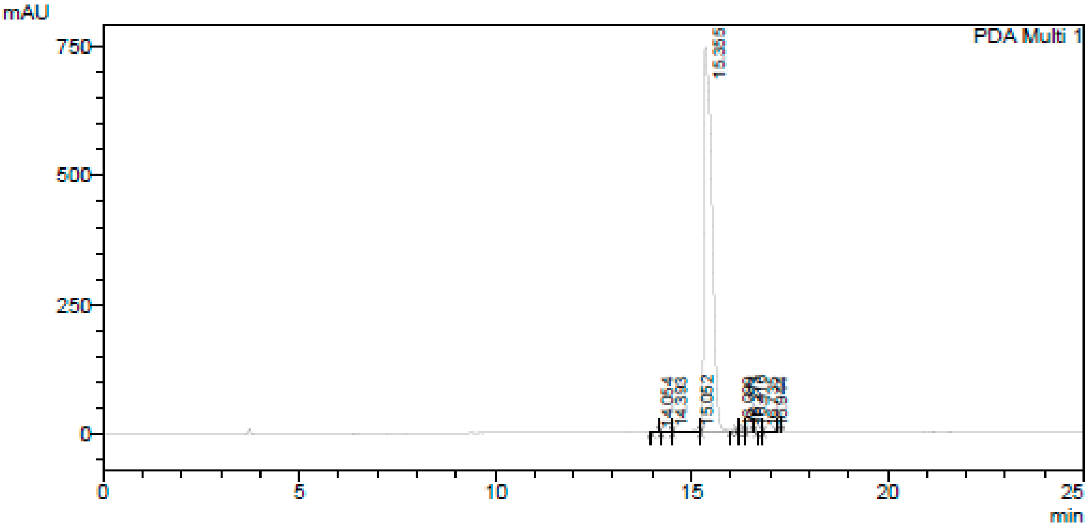

Figure S-2Lb. HPLC chromatogram of compound **2L** at 220 nm.

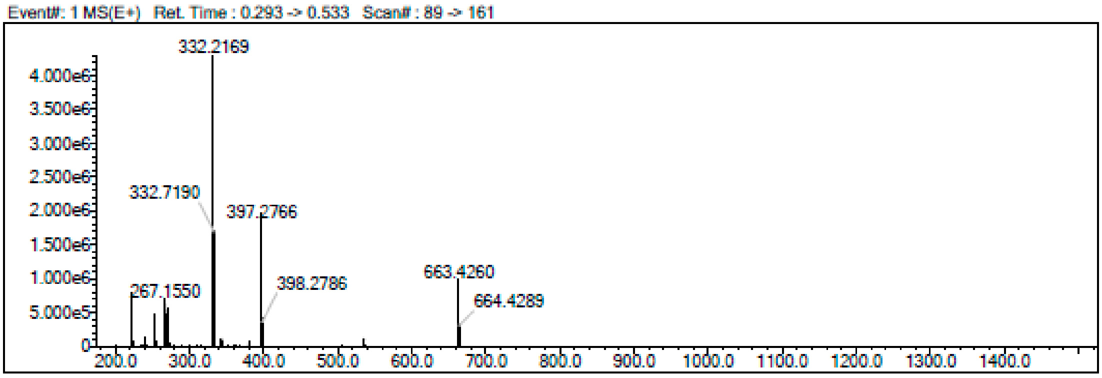

Figure S-2Lc. MS analysis of compound **2L**.

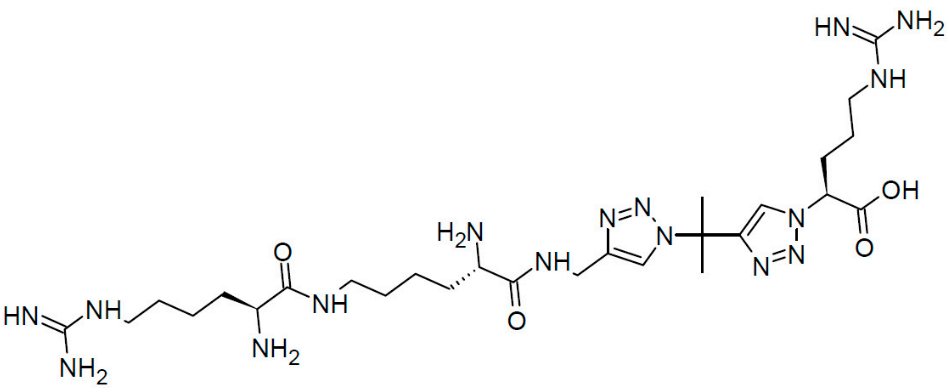

Figure S-2Da. Structure of compound **2D** H-D-Lys(Har)-Gly[Trl]Mba[Trl]Arg-OH.

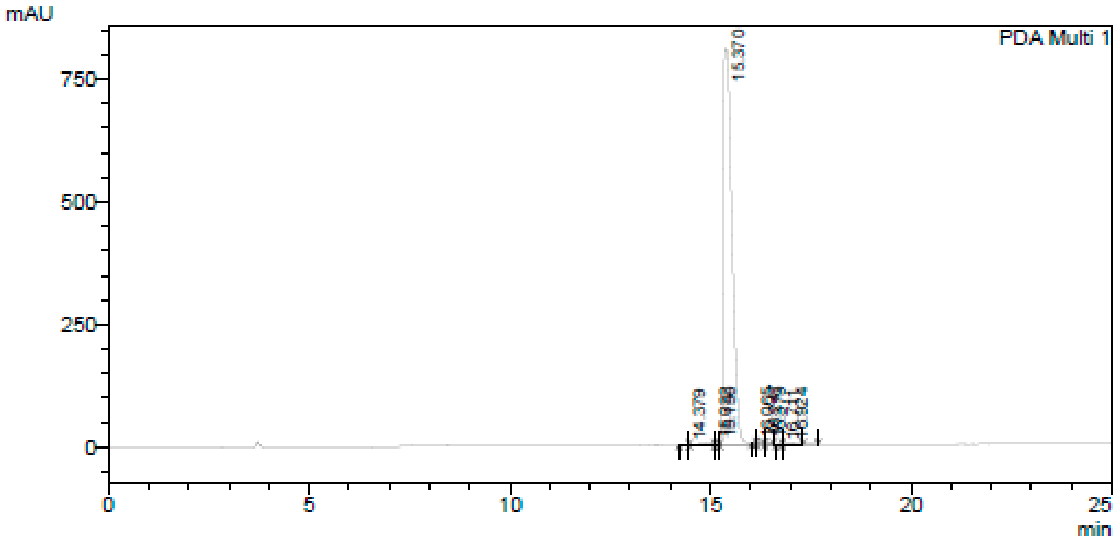

Figure S-2Db. HPLC chromatogram of compound **2D** at 220 nm.

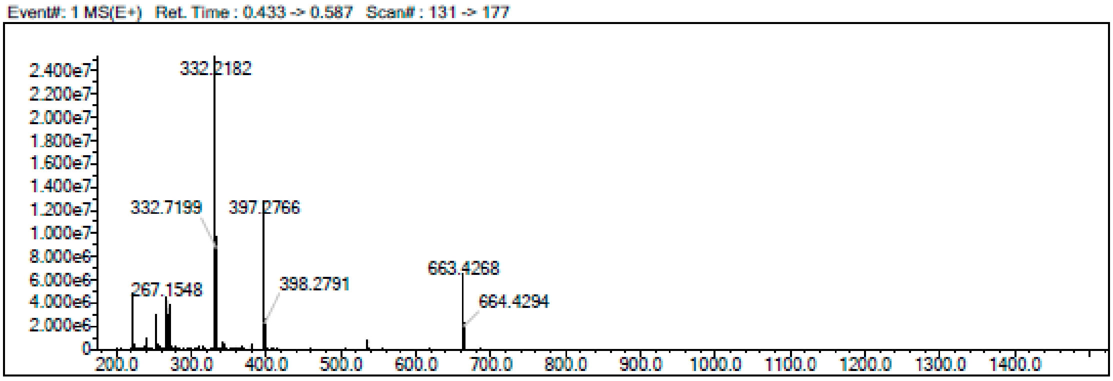

Figure S-2Dc. MS analysis of compound **2D**.

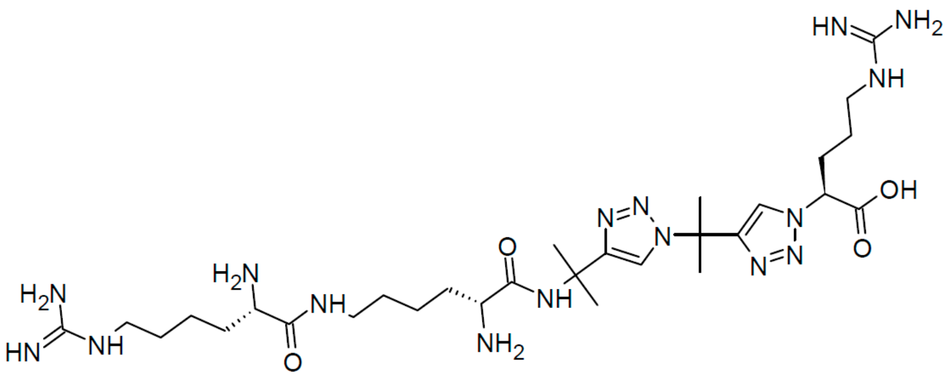

Figure S-3La. Structure of compound **3L** H-Lys(Har)-Mba[Trl]Mba[Trl]Arg-OH.

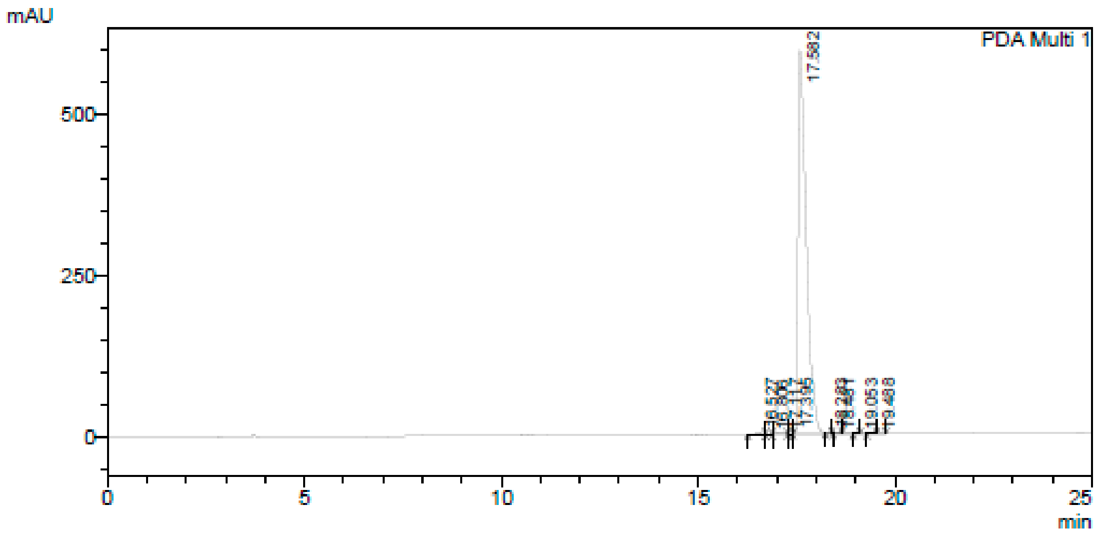

Figure S-3Lb. HPLC chromatogram of compound **3L** at 220 nm.

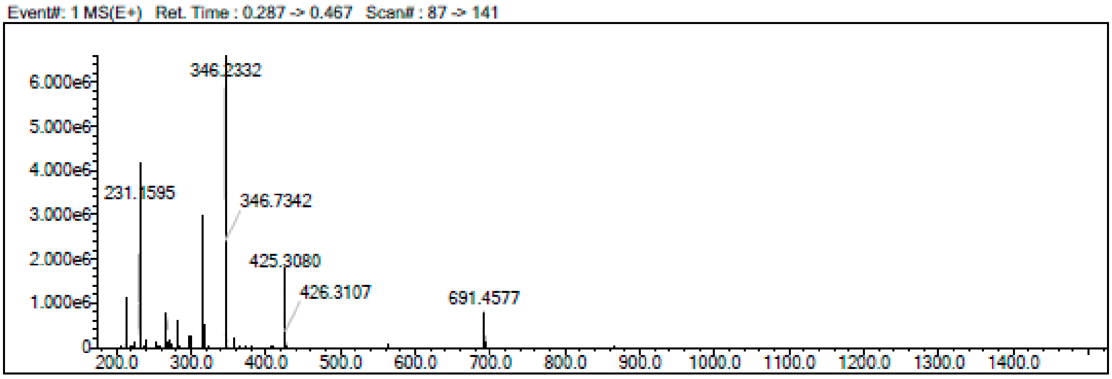

Figure S-3Lc. MS analysis of compound **3L**.

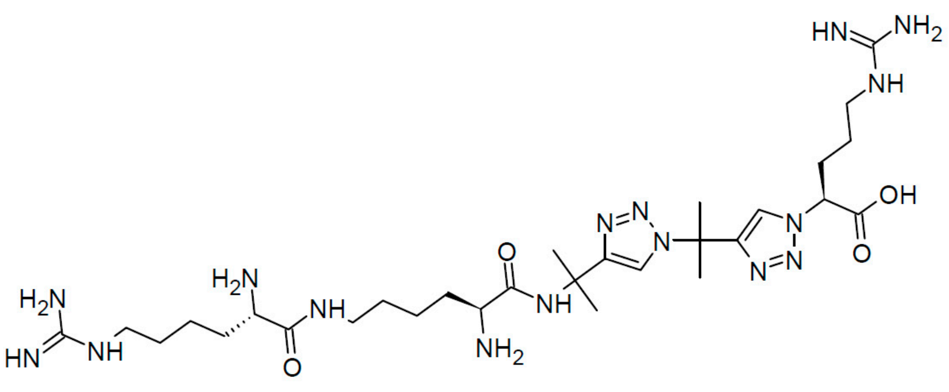

Figure S-3Da. Structure of compound **3D** H-D-Lys(Har)-Mba[Trl]Mba[Trl]Arg-OH.

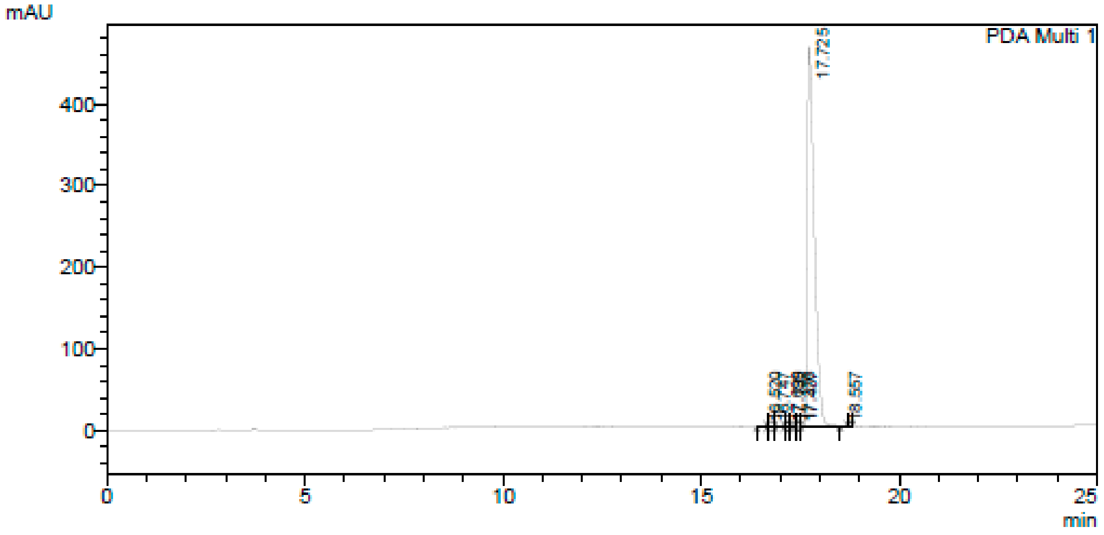

Figure S-3Db. HPLC chromatogram of compound **3D** at 220 nm.

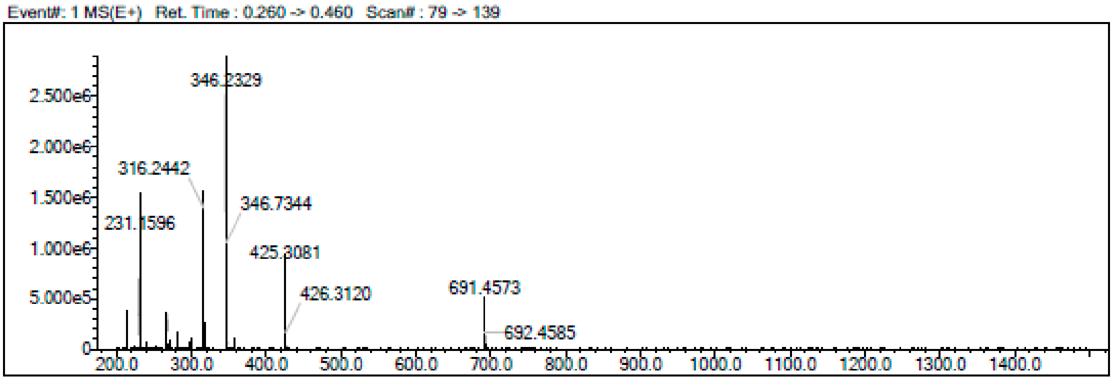

Figure S-3Dc. MS analysis of compound **3D**.

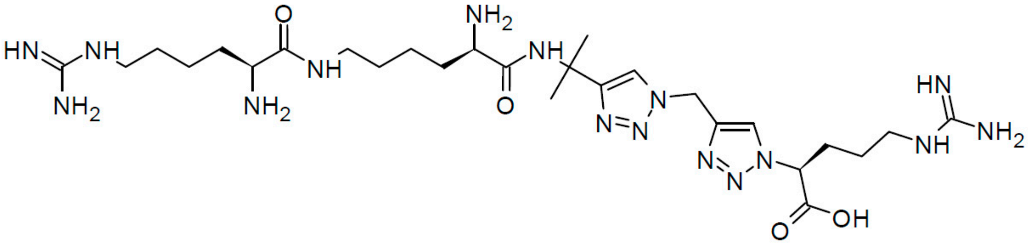

Figure S-4La. Structure of compound **4L** H-Lys(Har)-Mba[Trl]Gly[Trl]Arg-OH.

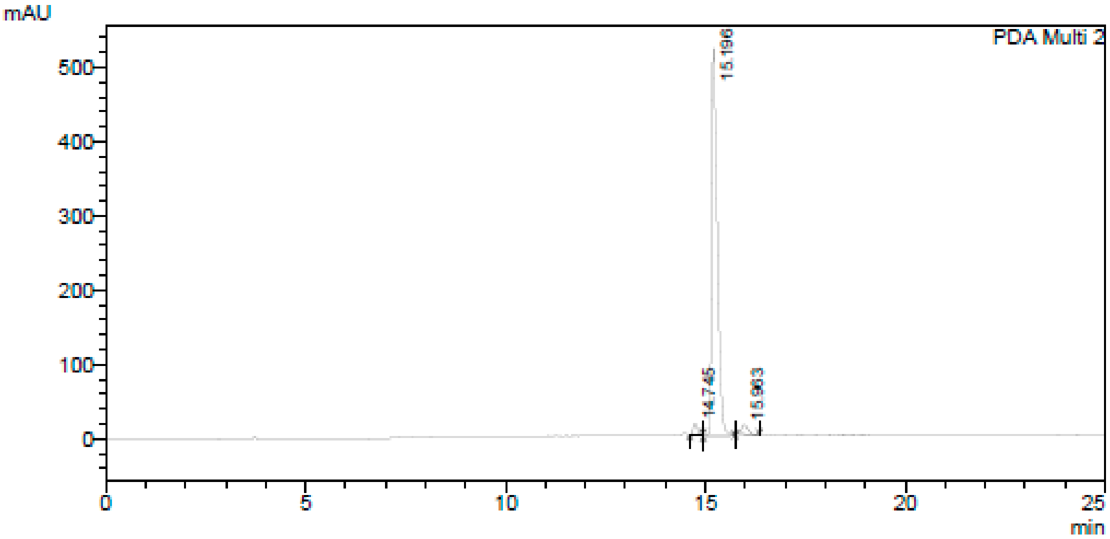

Figure S-4Lb. HPLC chromatogram of compound **4L** at 220 nm.

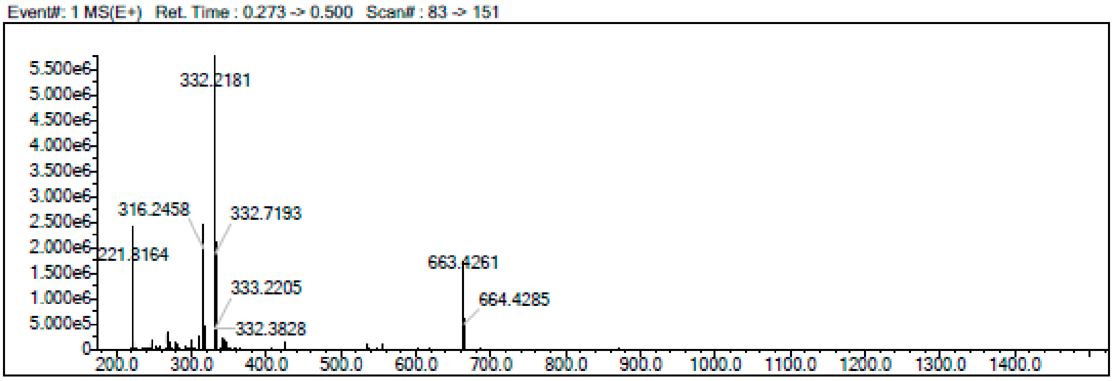

Figure S-4Lc. MS analysis of compound 4**L**.

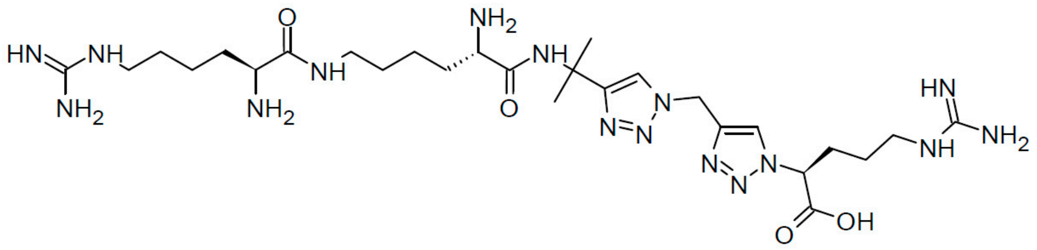

Figure S-4Da. Structure of compound **4D** H-D-Lys(Har)-Mba[Trl]Gly[Trl]Arg-OH.

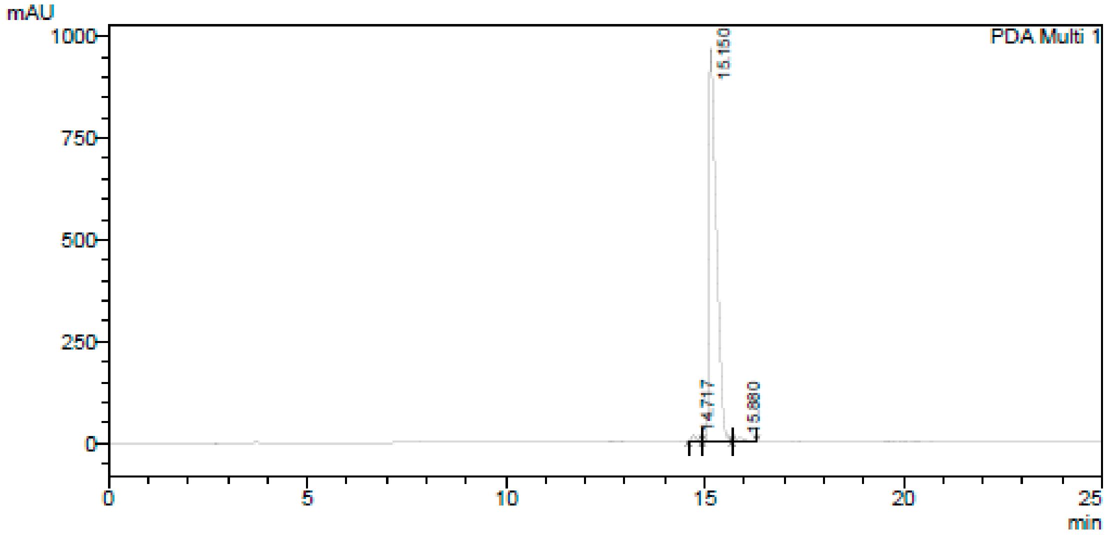

Figure S-4Db. HPLC chromatogram of compound **4D** at 220 nm.

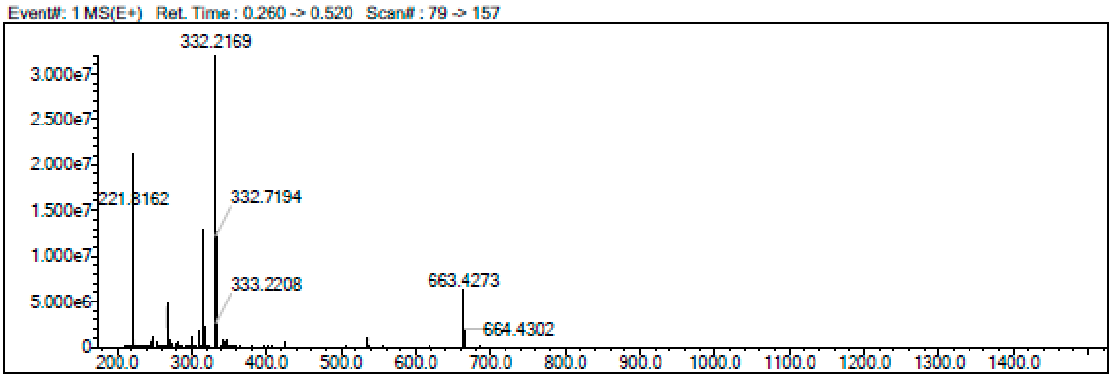

Figure S-4Dc. MS analysis of compound 4**D**.

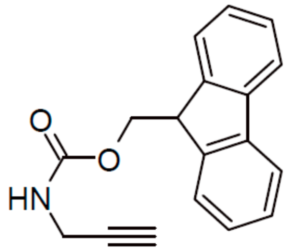

Figure S-5a. Structure of compound **5** Fmoc-propargylamine.

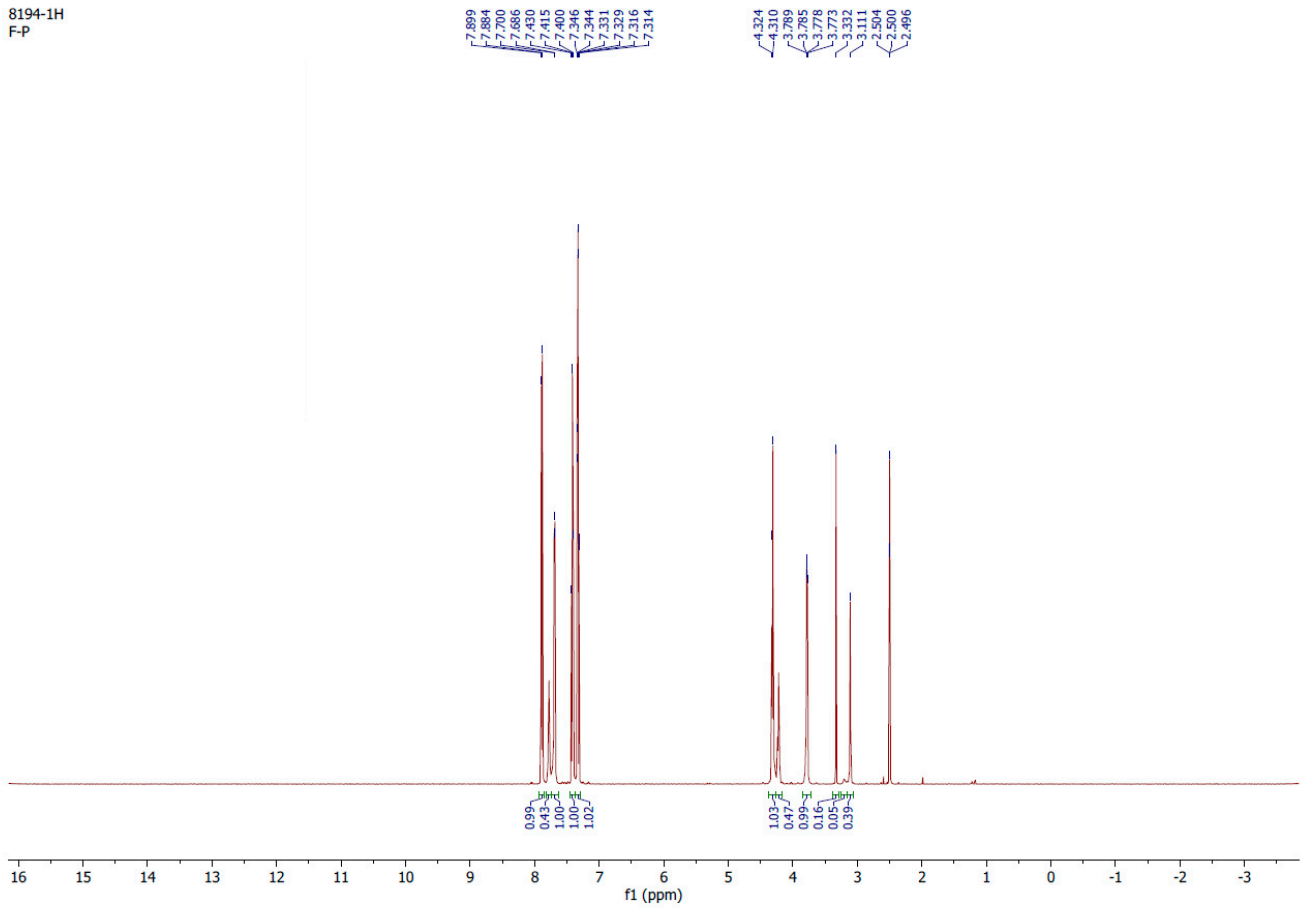

Figure S-5b. ^{1}H NMR spectrum of compound **5** Fmoc-propargylamine.

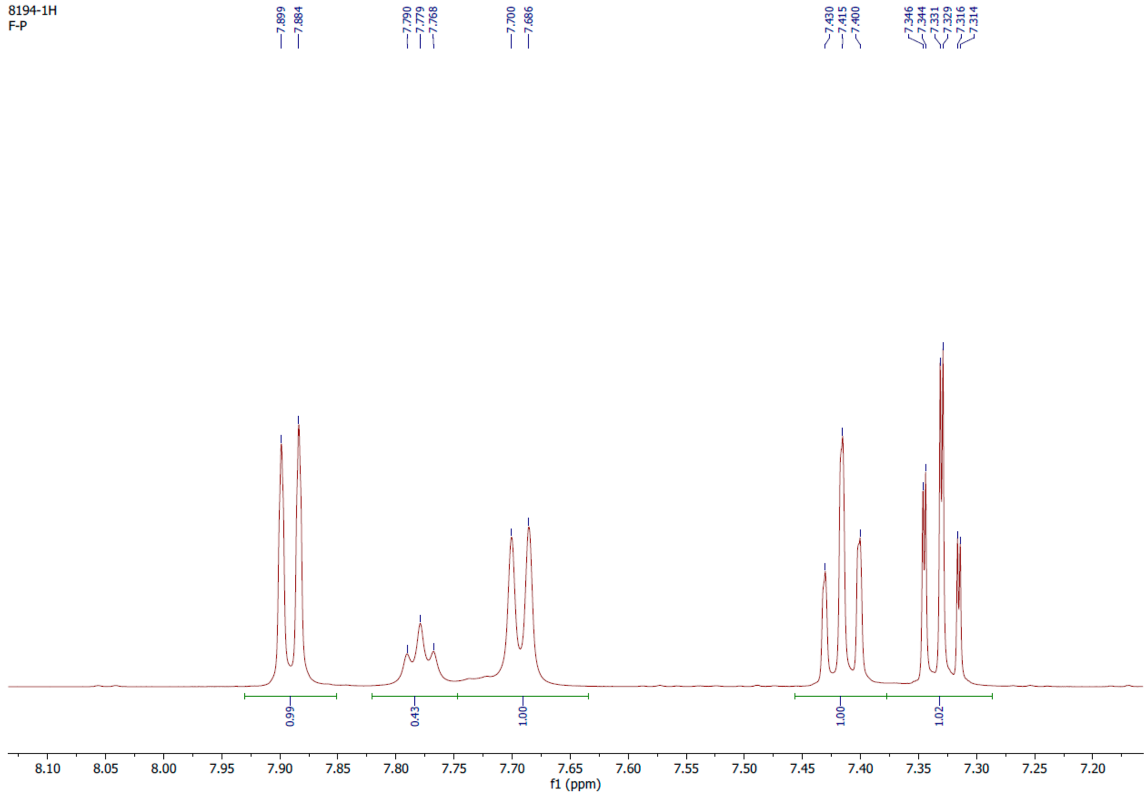

Figure S-5c. ^{1}H NMR spectrum of compound **5** Fmoc-propargylamine, zoom 7.20 – 8.10 ppm.

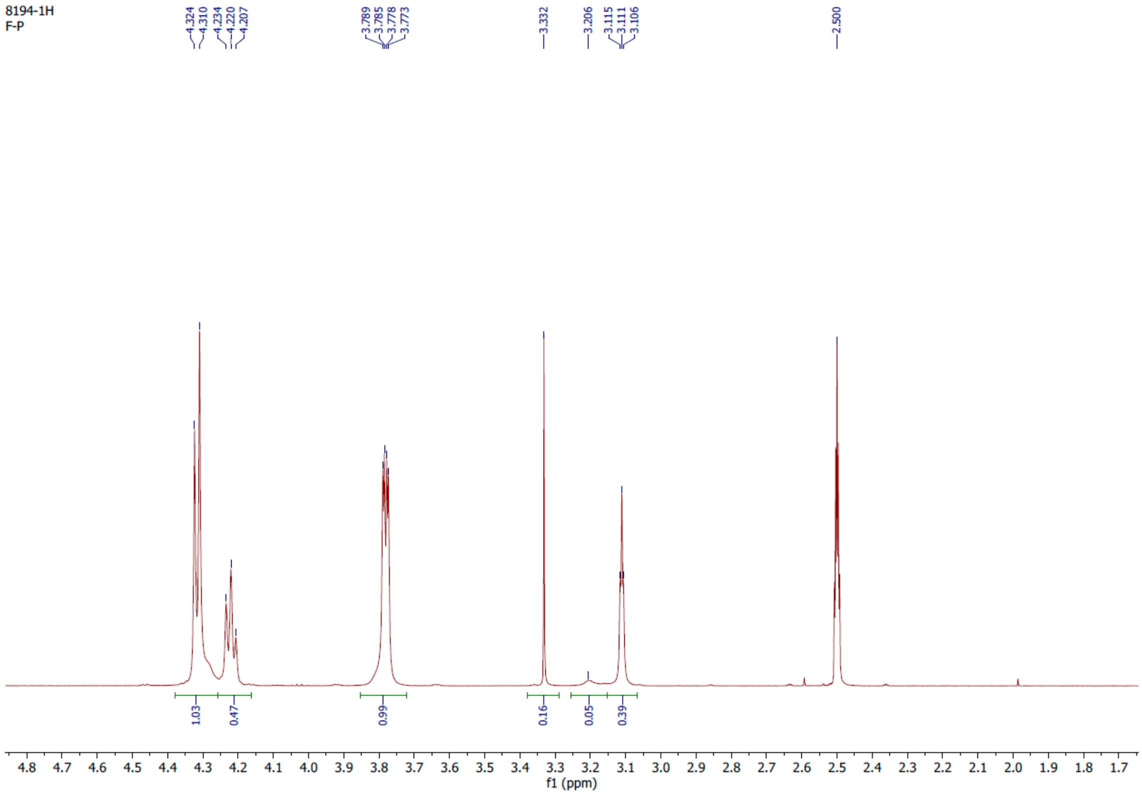

Figure S-5d. ^{1}H NMR spectrum of compound **5** Fmoc-propargylamine, zoom 1.70 – 4.80 ppm.

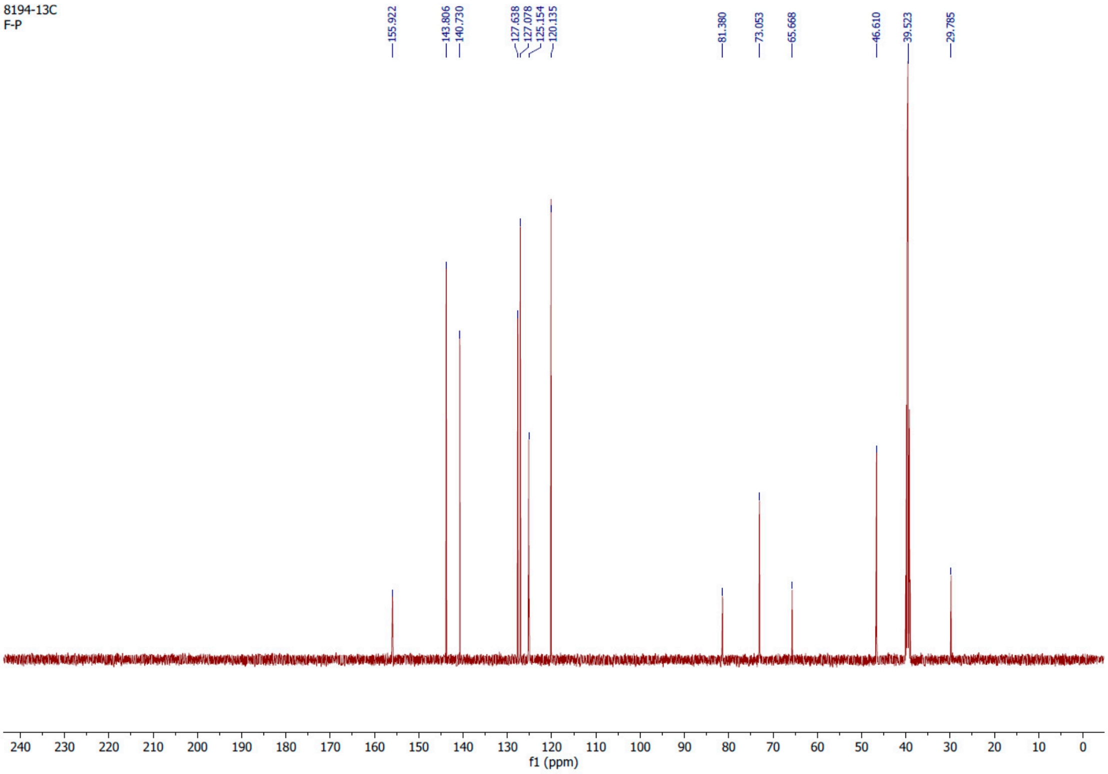

Figure S-5e. ^{13}C NMR spectrum of compound **5** Fmoc-propargylamine.

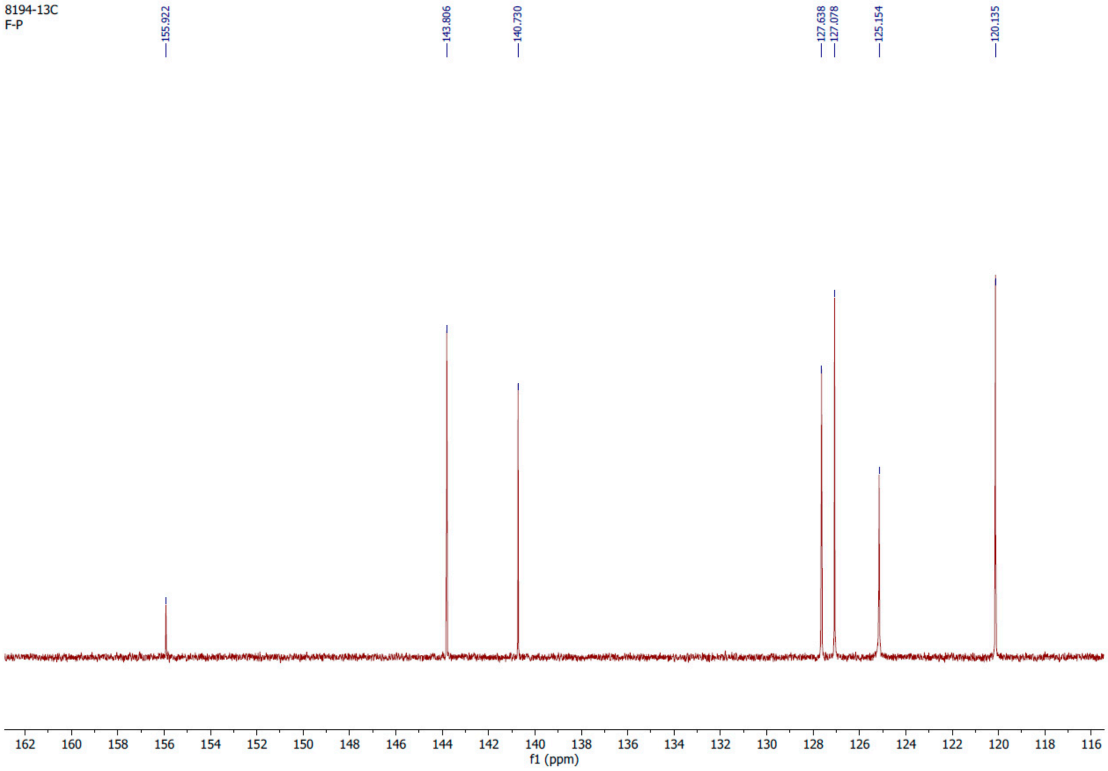

Figure S-5f. ^{13}C NMR spectrum of compound **5** Fmoc-propargylamine, zoom 116 – 162 ppm.

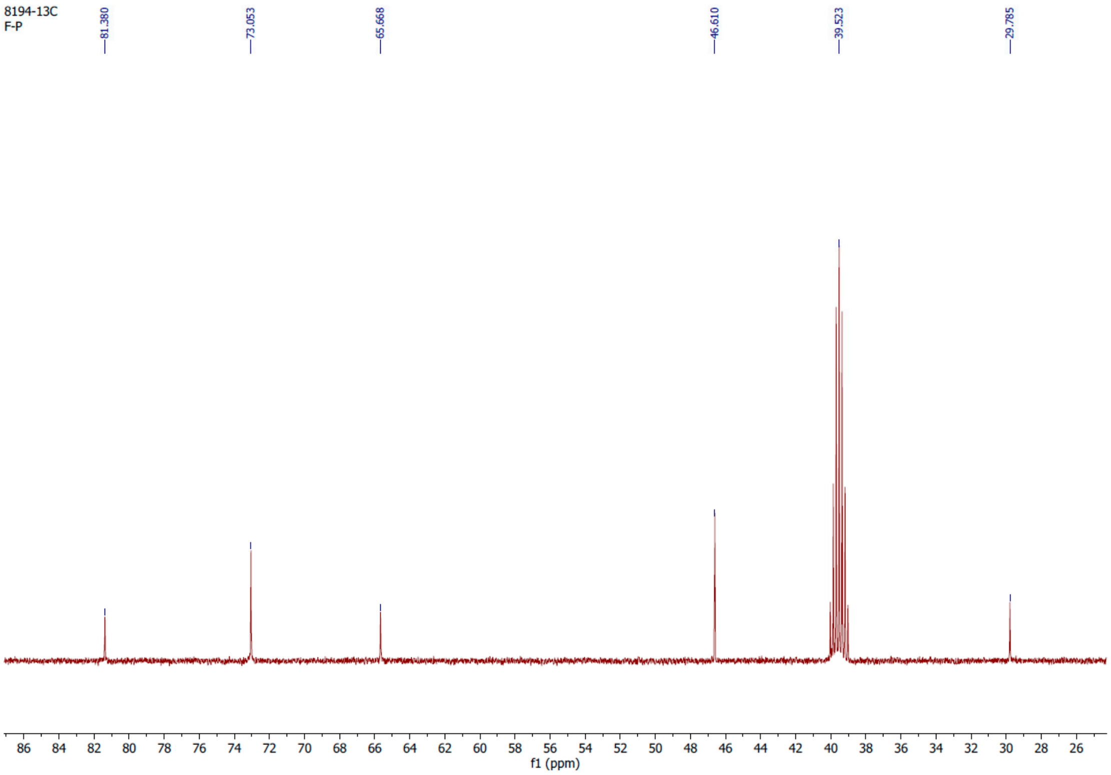

Figure S-5g. ^{13}C NMR spectrum of compound **5** Fmoc-propargylamine, zoom 26 – 86 ppm.

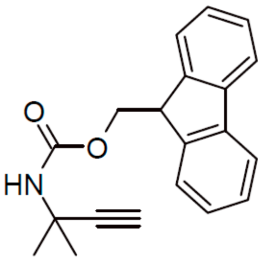

Figure S-6a. Structure of compound **6** Fmoc-Mba.

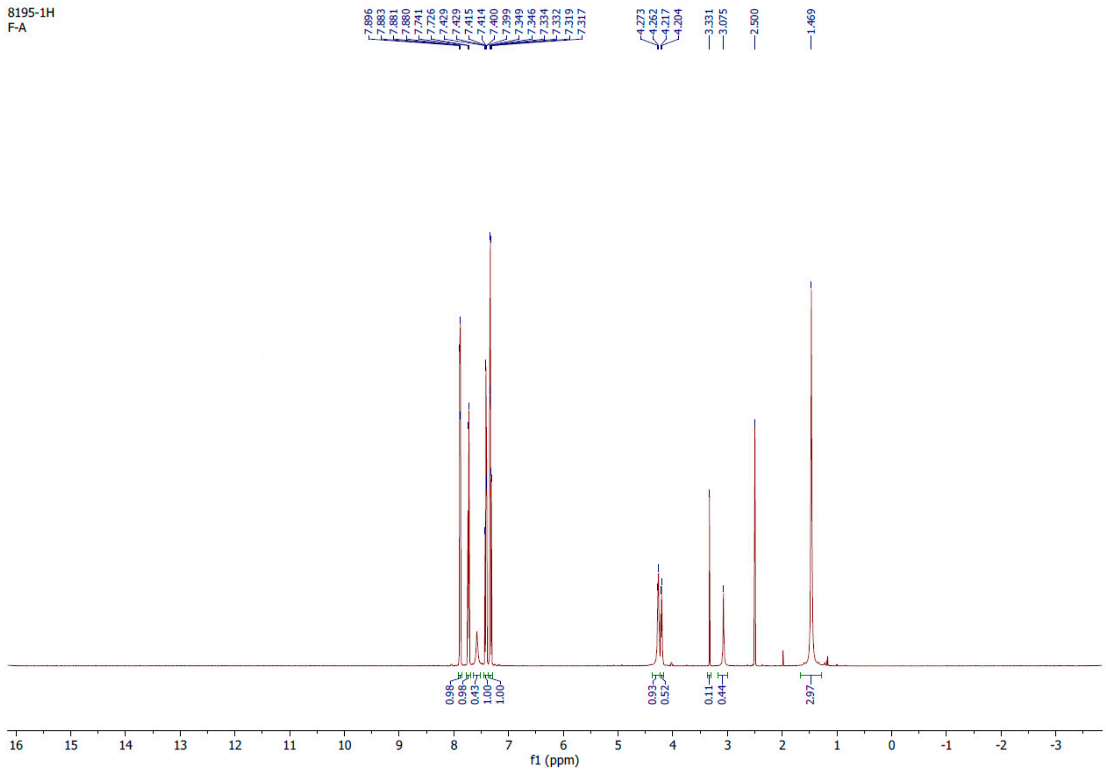

Figure S-6b. ^{1}H NMR spectrum of compound **6** Fmoc-Mba.

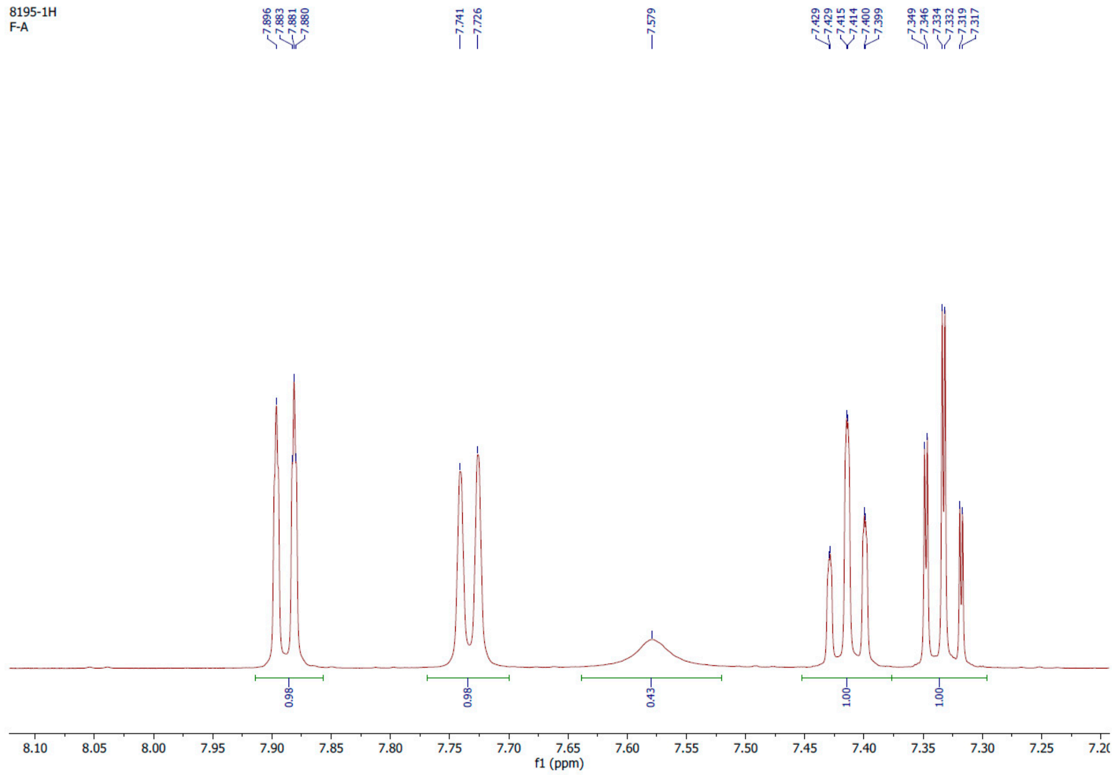

Figure S-6c. ^{1}H NMR spectrum of compound **6** Fmoc-Mba, zoom 7.20 – 8.10 ppm.

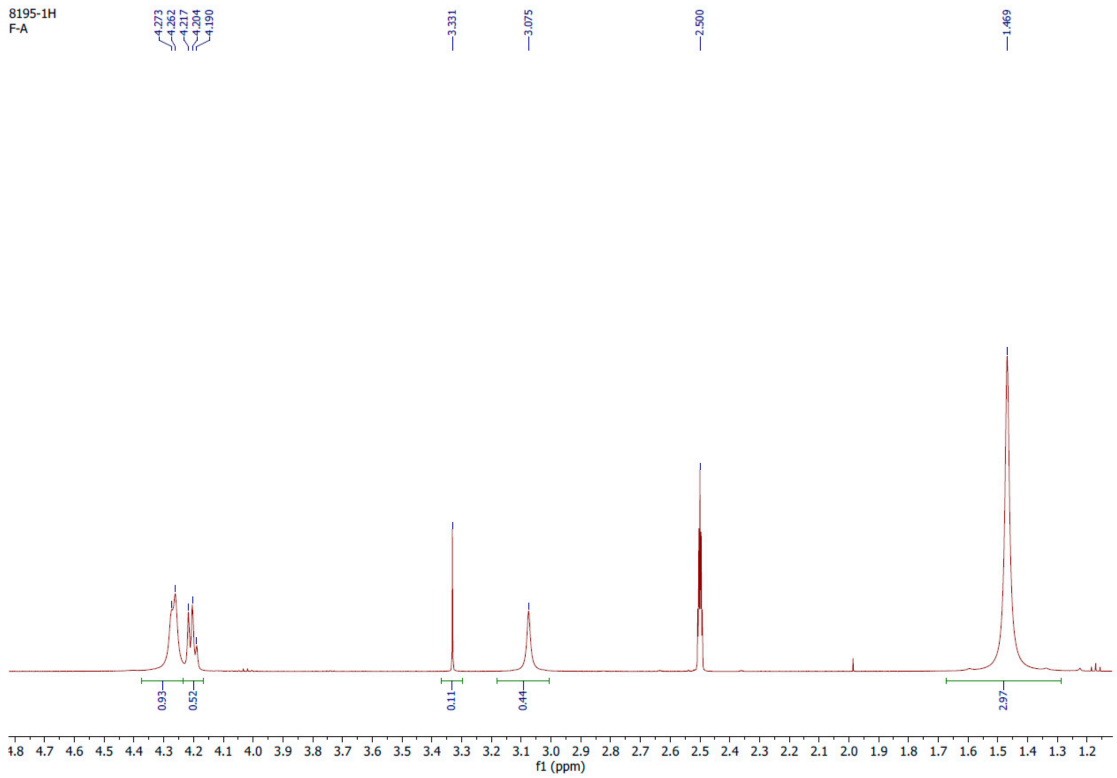

Figure S-6d. ^{1}H NMR spectrum of compound **6** Fmoc-Mba, zoom 1.70 – 4.80 ppm.

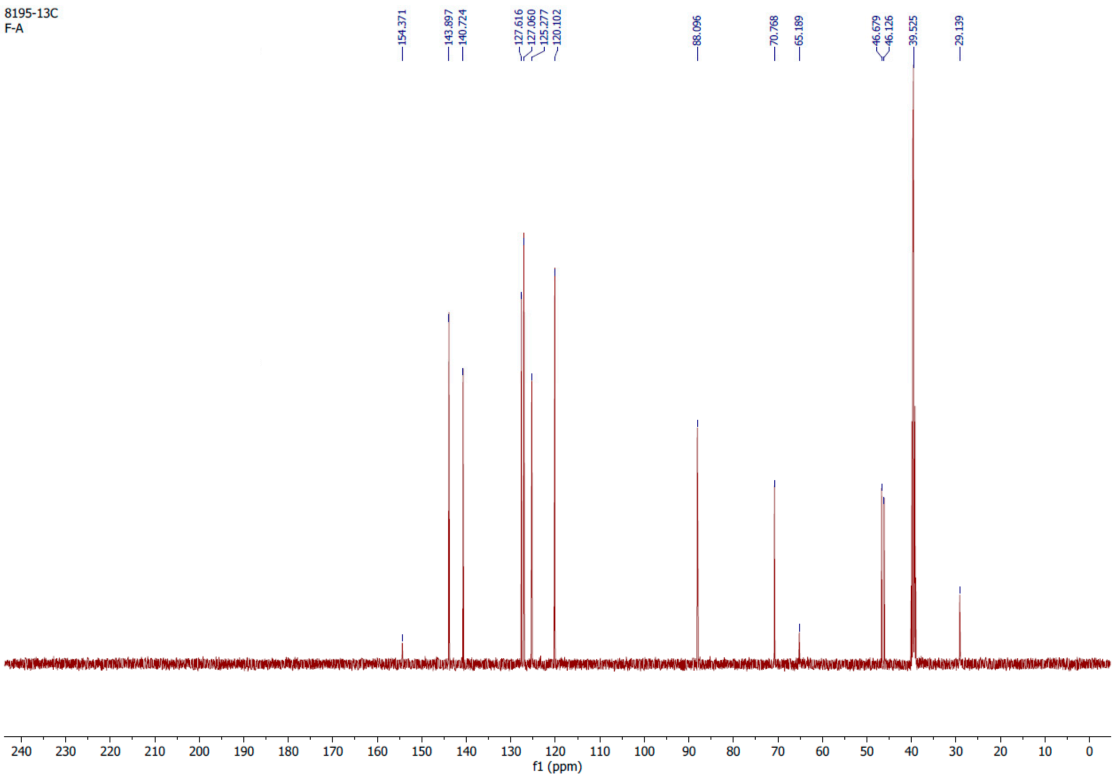

Figure S-6e. ^{13}C NMR spectrum of compound **6** Fmoc-Mba.

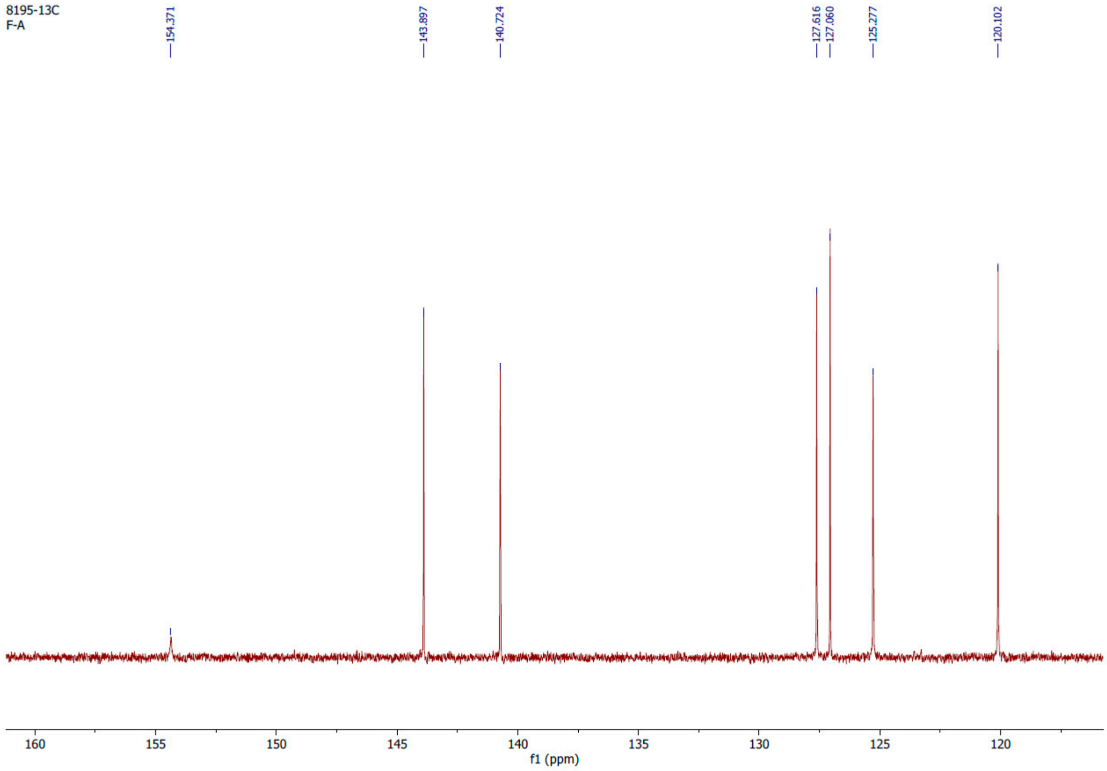

Figure S-6f. ^{13}C NMR spectrum of compound **6** Fmoc-Mba, zoom 116 – 162 ppm.

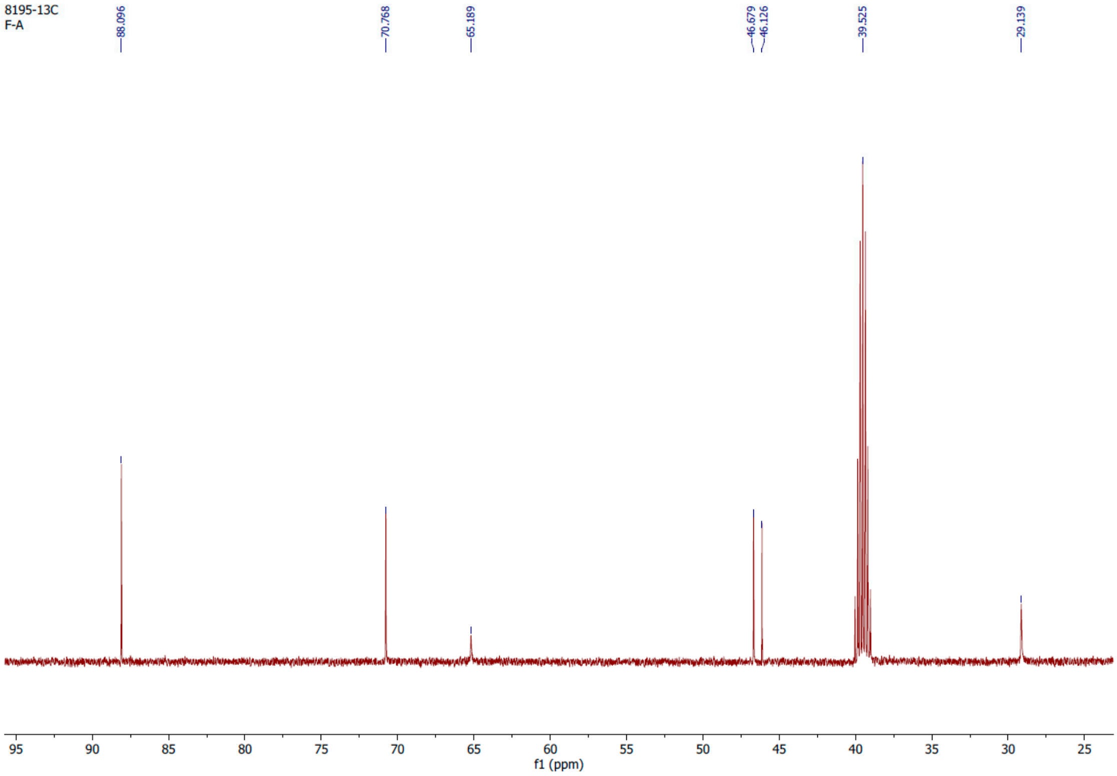

Figure S-6g. ^{13}C NMR spectrum of compound **6** Fmoc-Mba, zoom 26 – 86 ppm.

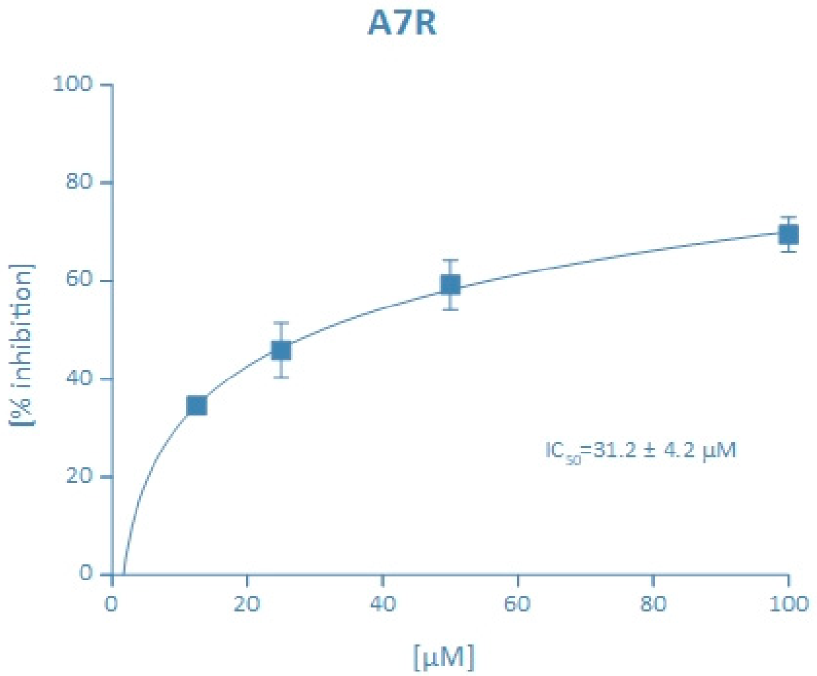

Figure S-7-A7R. Inhibition of VEGF 165A binding to human NRP-1 by A7R peptide. Inhibition was measured at 12,5µM, 25 µM, 50 µM and 100 µM. Experiments for every concentration were repeated three times. Mean of those are presented in the graphs together with standard deviation.

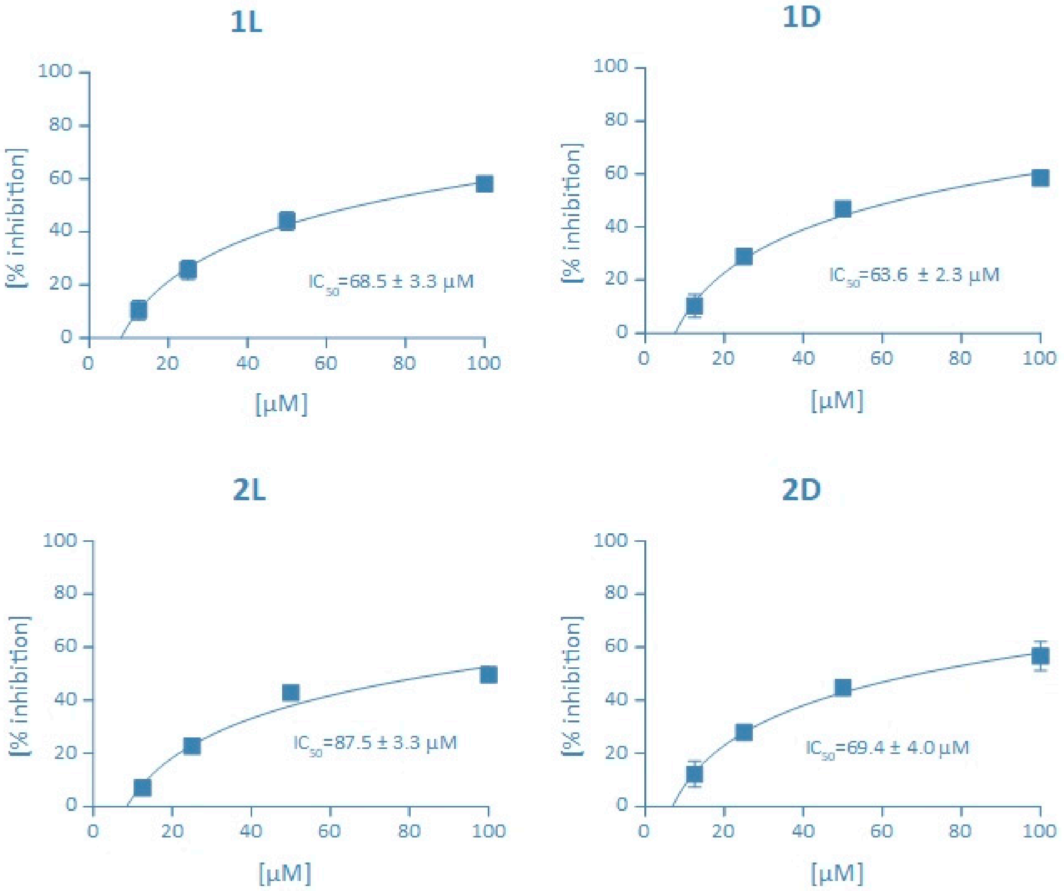

Figure S-7-1-2. Inhibition of VEGF 165A binding to human NRP-1 by different compounds. Inhibition by each compound was measured at 12,5µM, 25 µM, 50 µM and 100 µM. Experiments for every concentration were repeated three times. Mean of those are presented in the graphs together with standard deviation (usually not visible in this scale).

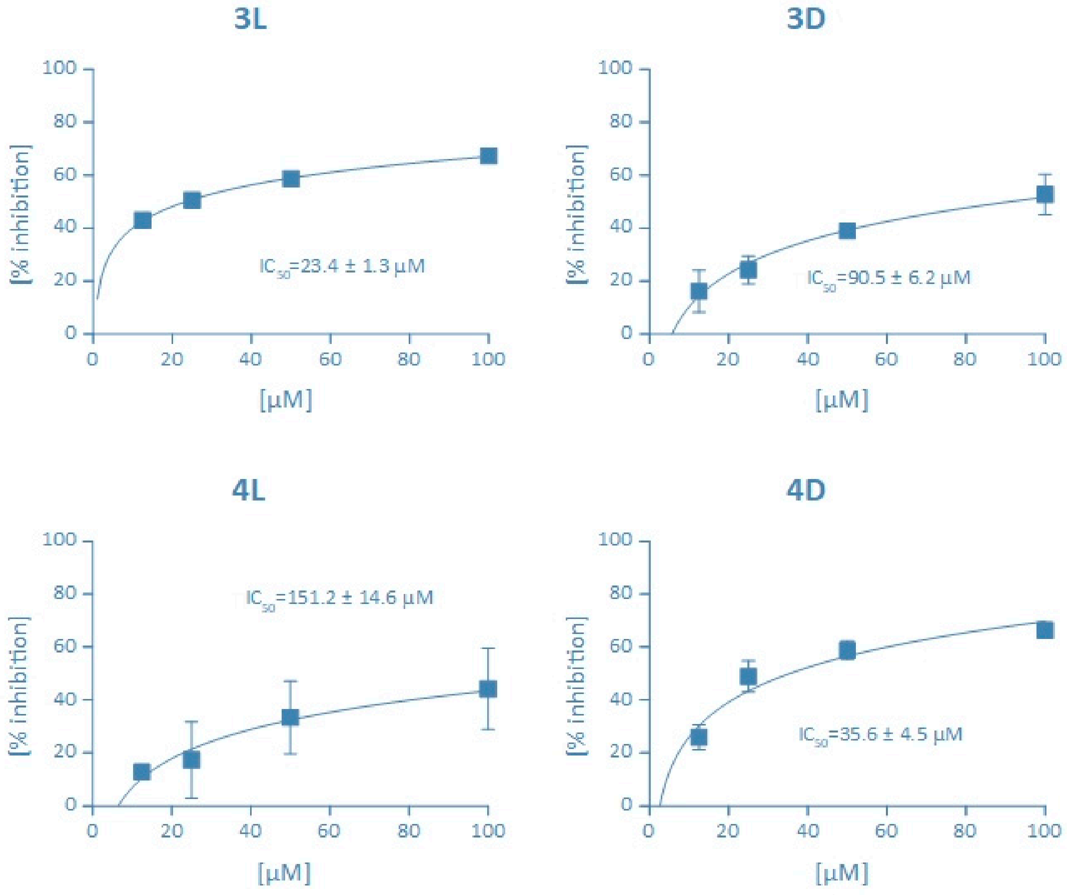

Figure S-7-3-4. Inhibition of VEGF 165A binding to human NRP-1 by different compounds. Inhibition by each compound was measured at 12,5μM, 25 μM, 50 μM and 100 μM. Experiments for every concentration were repeated three times, except for compounds 3L and 4L where third measurement was divergent and not taken into account. Mean of those measurements are presented in the graphs together with standard deviation (usually not visible in this scale).

Table S-1. Energies obtained in Molecular Modeling of complex formation of **1L,D-4L,D** compounds with Neuropilin-1 protein.

compound	Energy after minimalization [kJ/mol]	Binding energy (H_2O)	Binding energy (no H_2O)	Relative binding energy [%]
**1L**	-644453	3095.67	1288.76	100.0
**1D**	-644681	2930.96	1238.61	96.1
**2L**	-644505	3051.34	1292.47	100.3
**2D**	-644216	3014.06	1306.81	101.4
**3L**	-644392	3162.33	1357.45	105.3
**3D**	-644676	3190.63	1374.72	106.7
**4L**	-644048	3073.27	1305.43	101.3
**4D**	-644562	3057.57	1318.94	102.3

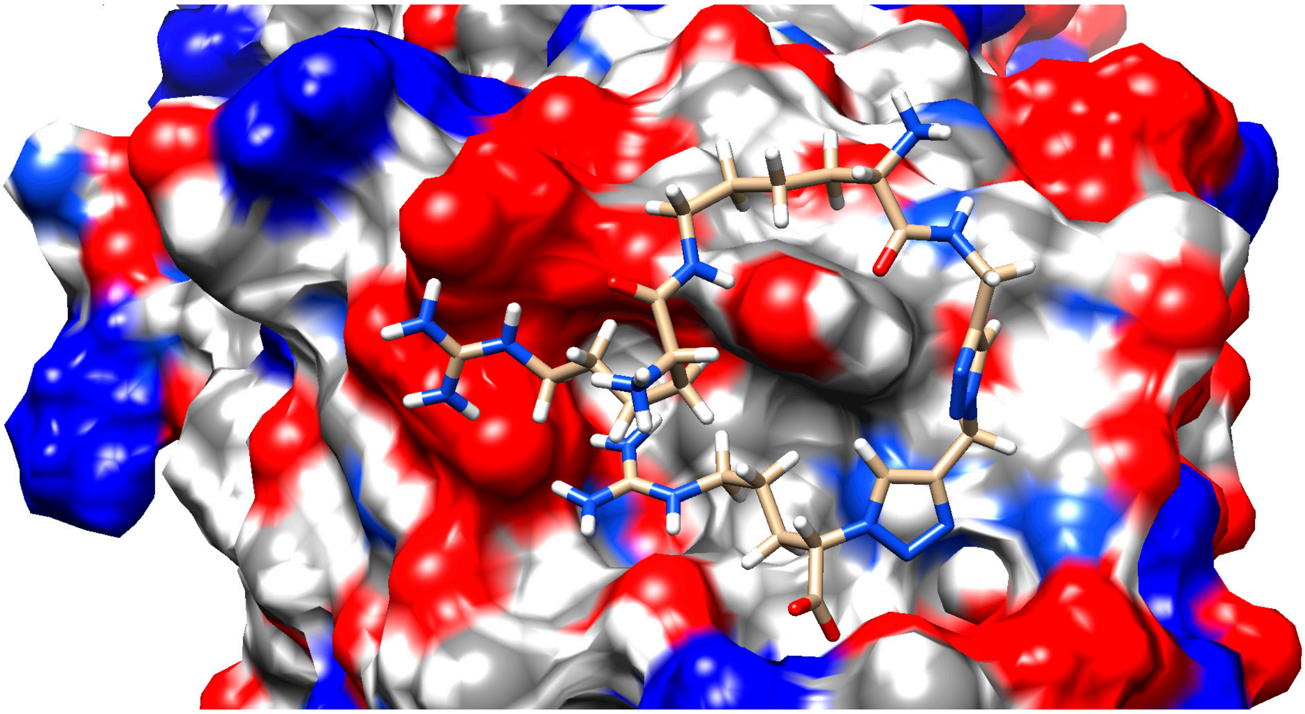

Figure S-8-1L. Complex of compound **1L** with NRP-1 after energy minimalization.

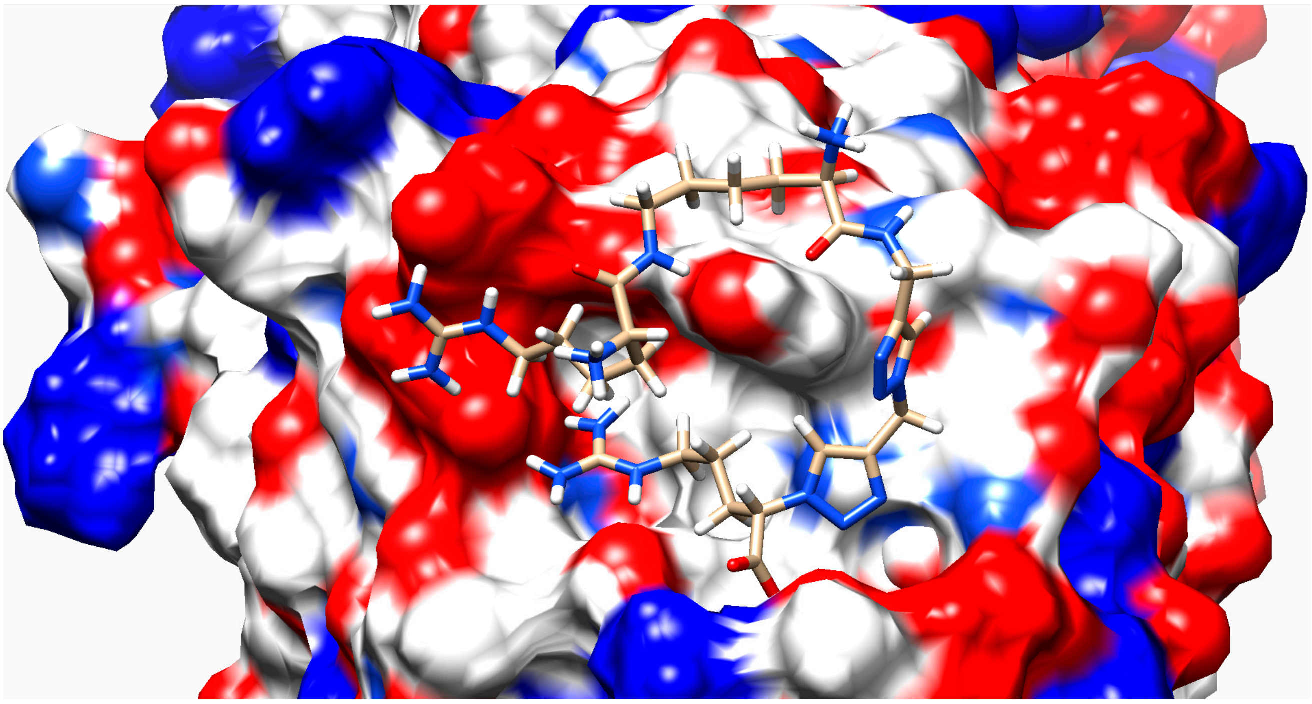

Figure S-8-1D. Complex of compound **1D** with NRP-1 after energy minimalization.

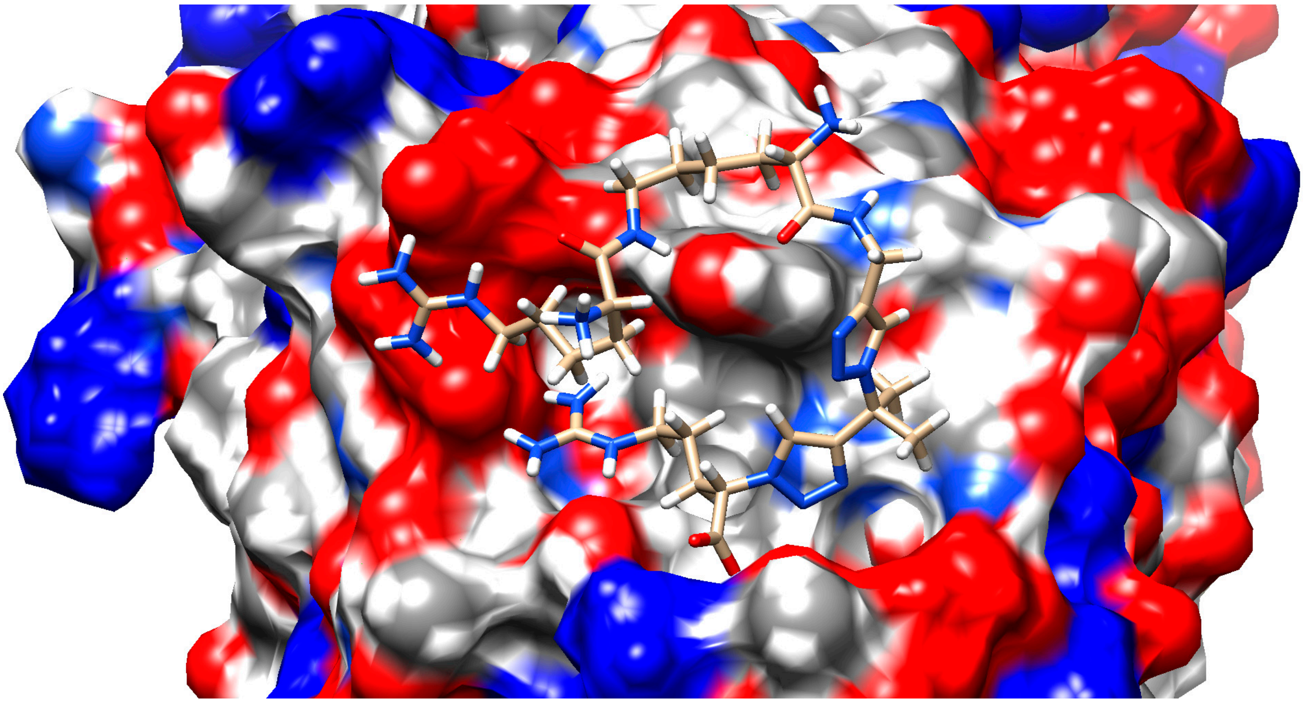

Figure S-8-2L. Complex of compound **2L** with NRP-1 after energy minimalization.

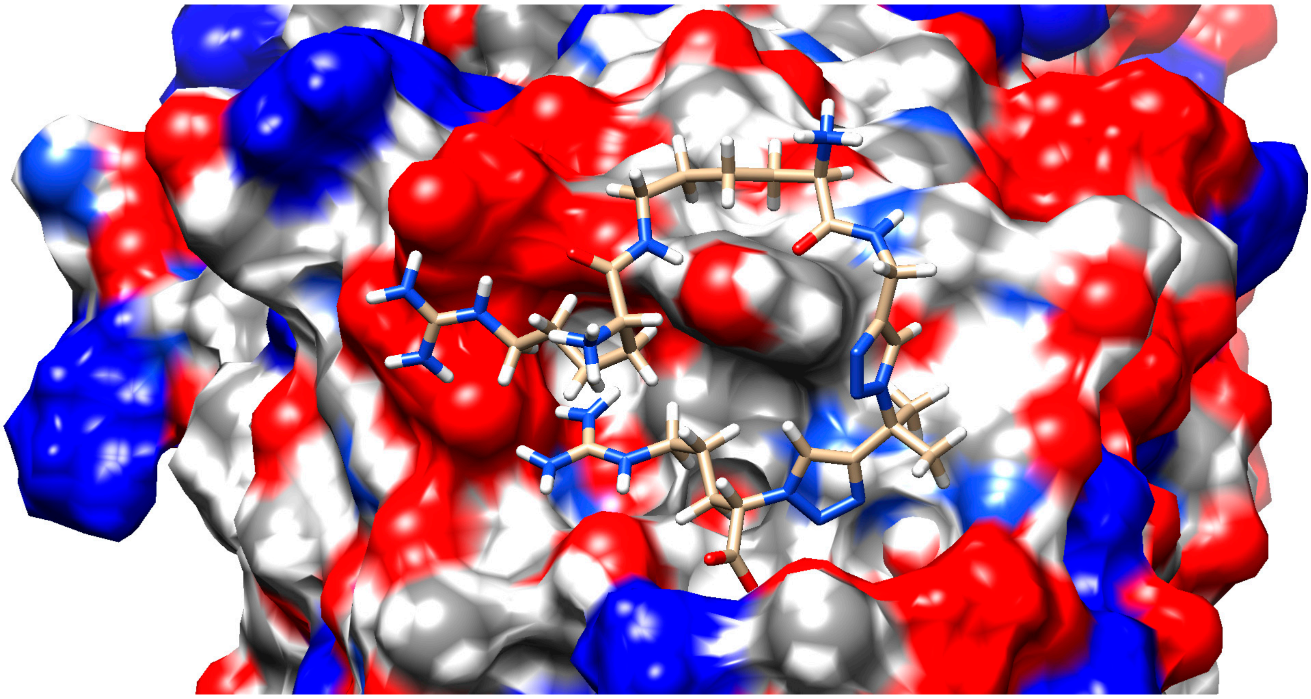

Figure S-8-2D. Complex of compound **2D** with NRP-1 after energy minimalization.

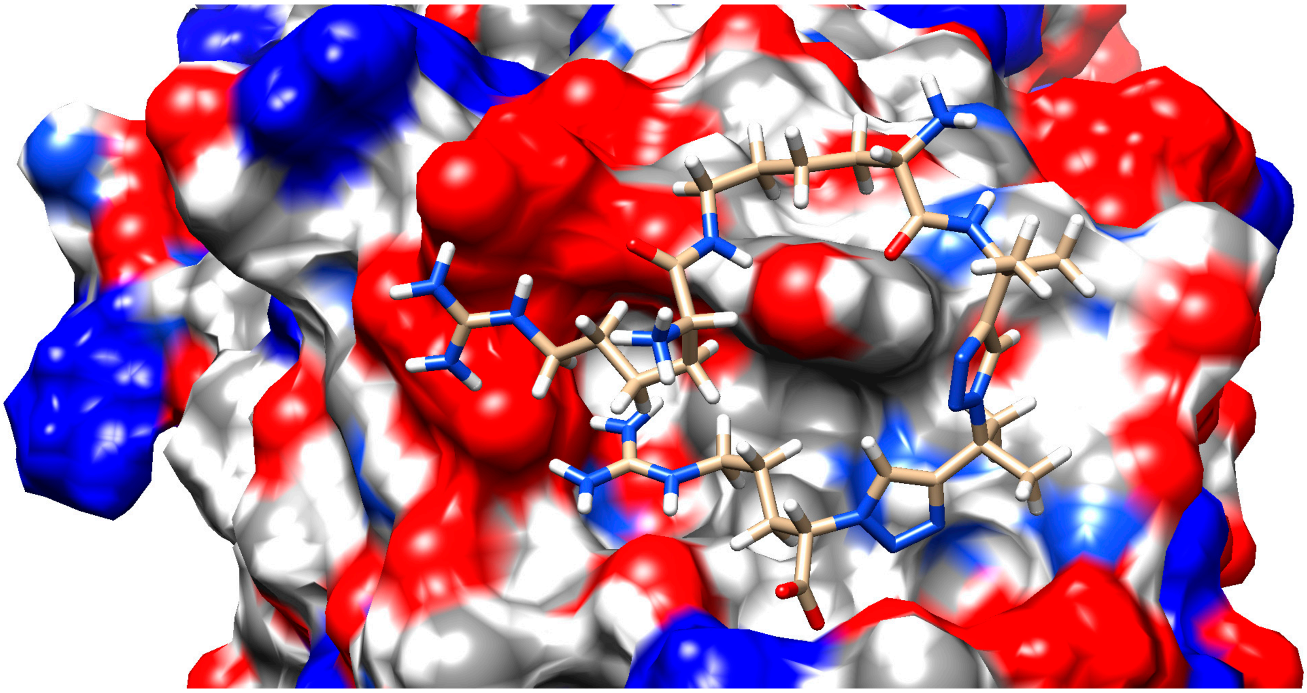

Figure S-8-3L. Complex of compound **3L** with NRP-1 after energy minimalization.

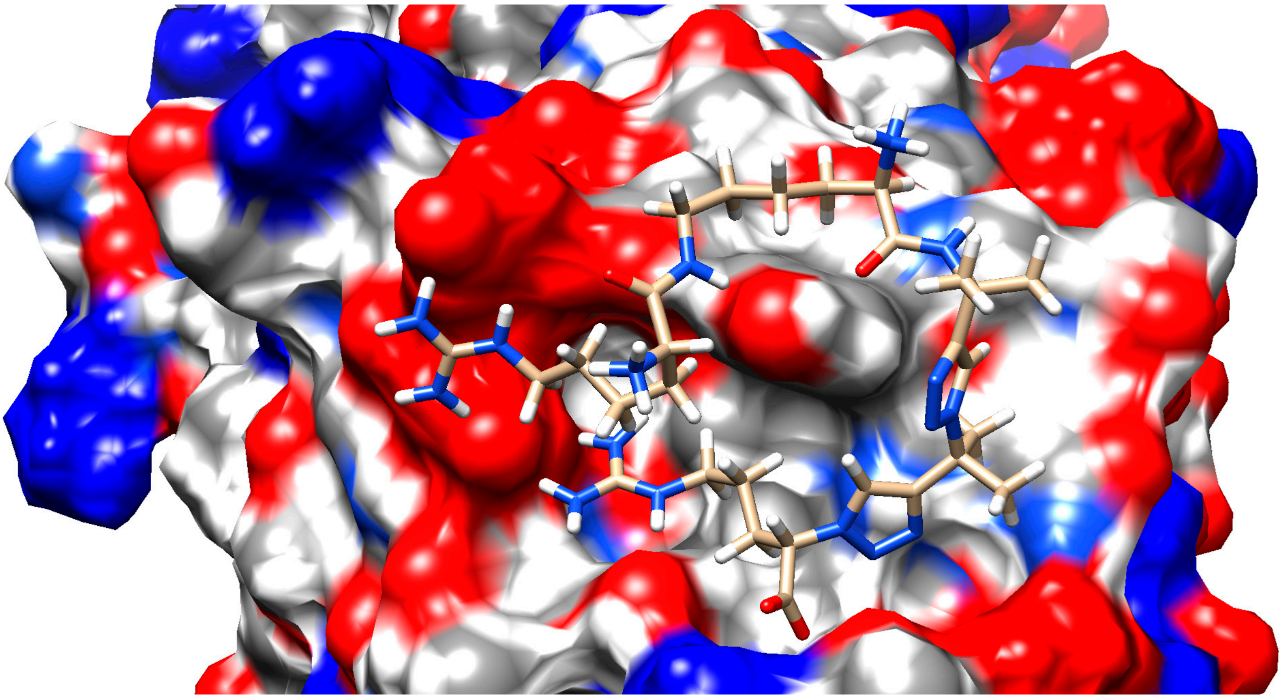

Figure S-8-3D. Complex of compound **3D** with NRP-1 after energy minimalization.

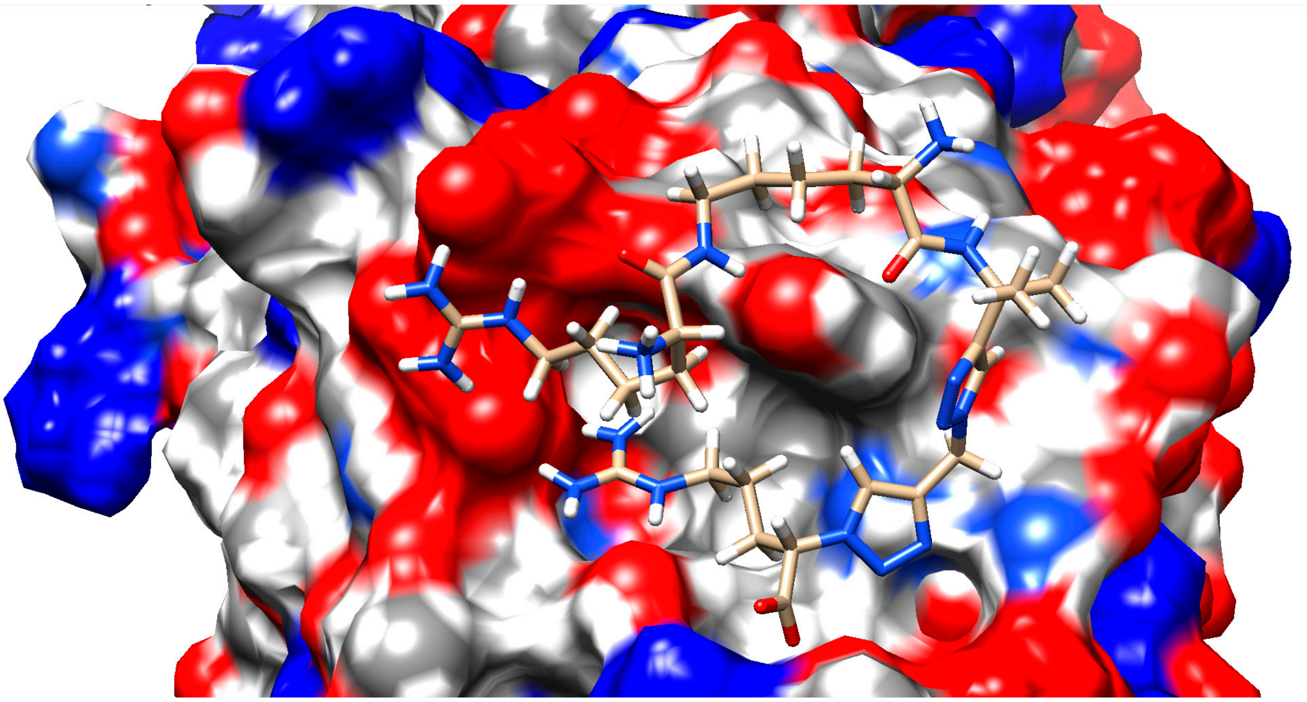

Figure S-8-4L. Complex of compound **4L** with NRP-1 after energy minimalization.

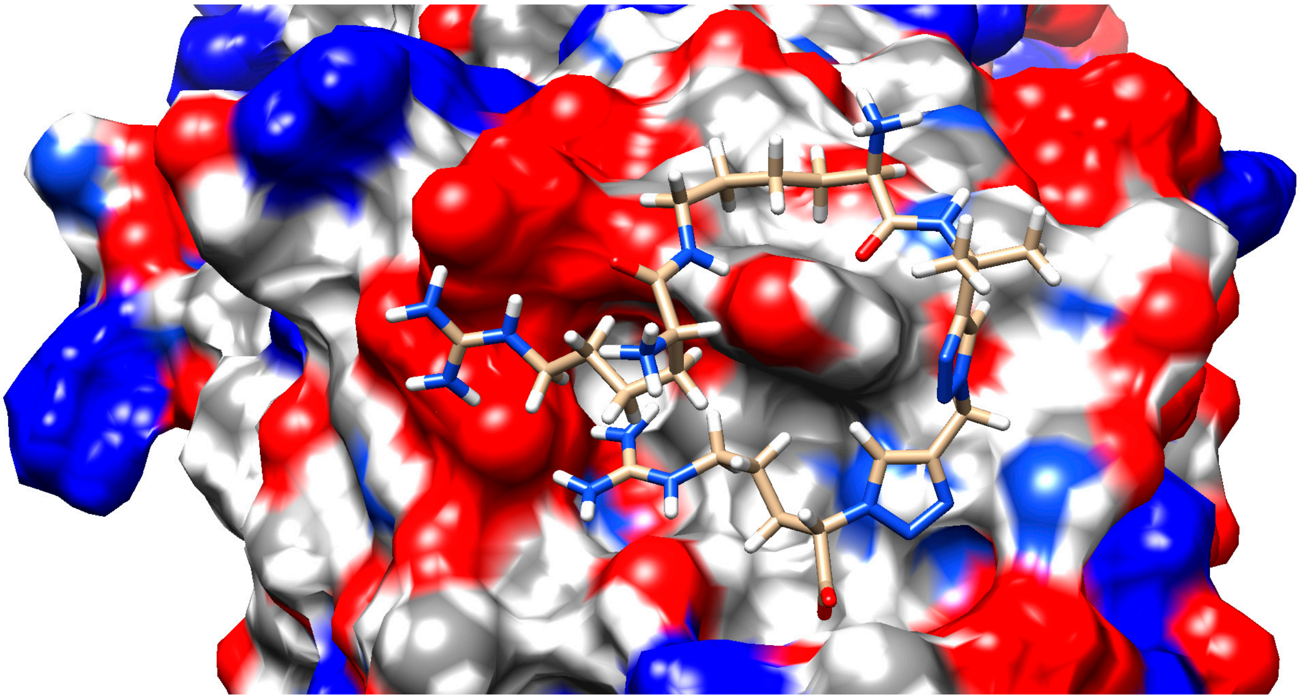

Figure S-8-4D. Complex of compound **4D** with NRP-1 after energy minimalization.

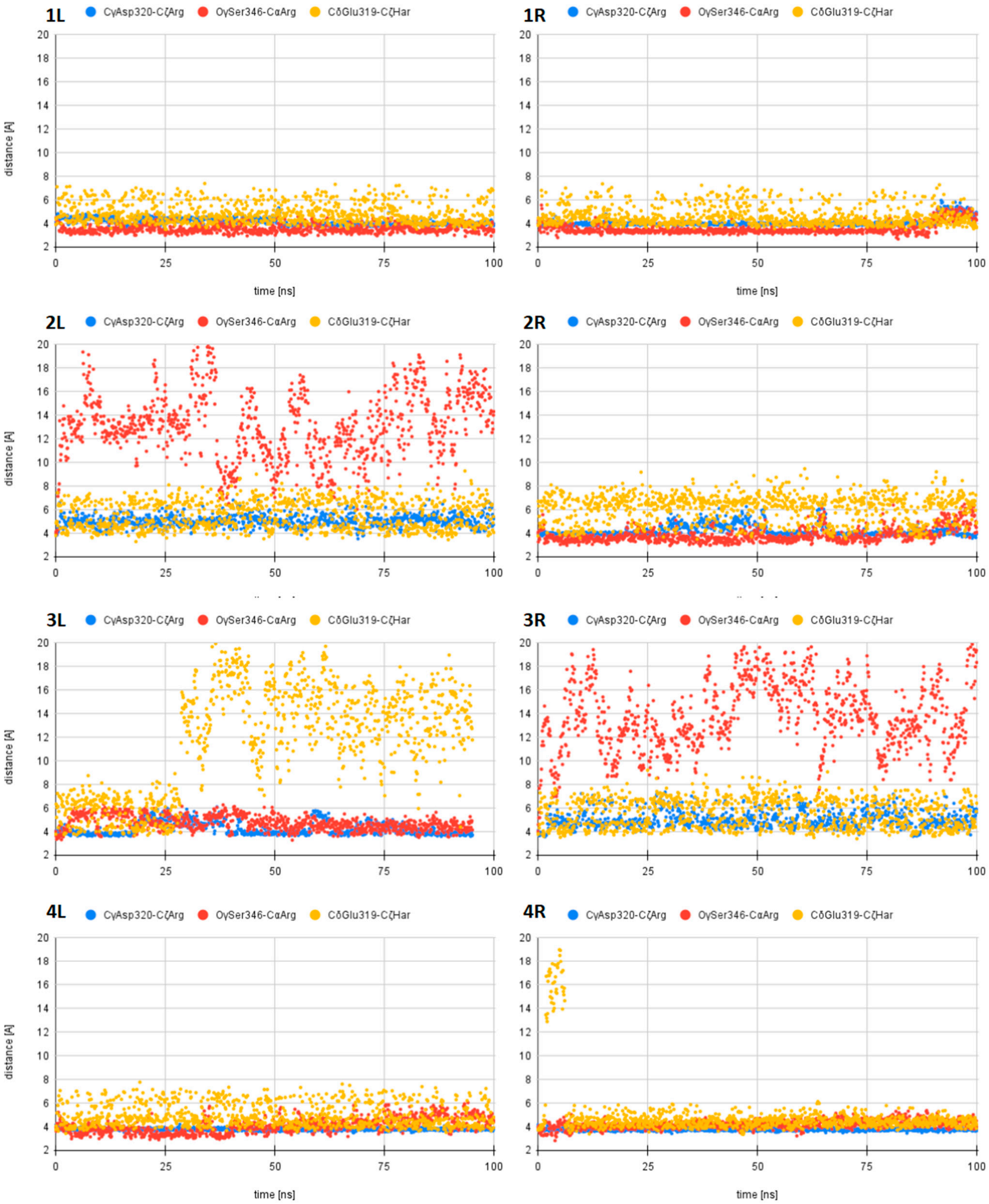

Figure S-9. Calculated distances, in Å, between atoms from the studied compounds and atoms of NRP-1; blue – CγAsp320-CζArg, red – OγSer346-CαArg, yellow – CδGlu319-CζHar; during 100 ns of simulation.
